# *ZAXINONE SYNTHASE 2* regulates growth and arbuscular mycorrhizal symbiosis in rice

**DOI:** 10.1093/plphys/kiac472

**Published:** 2022-10-12

**Authors:** Abdugaffor Ablazov, Cristina Votta, Valentina Fiorilli, Jian You Wang, Fatimah Aljedaani, Muhammad Jamil, Aparna Balakrishna, Raffaella Balestrini, Kit Xi Liew, Chakravarthy Rajan, Lamis Berqdar, Ikram Blilou, Luisa Lanfranco, Salim Al-Babili

**Affiliations:** Biological and Environmental Sciences and Engineering Division, Center for Desert Agriculture (CDA), King Abdullah University of Science and Technology (KAUST), The BioActives Lab, Thuwal, 23955-15 6900, Saudi Arabia; The Plant Science Program, Biological and Environmental Science and Engineering Division, King Abdullah University of Science and Technology (KAUST), Thuwal, Saudi Arabia; Department of Life Sciences and Systems Biology, University of Torino, Torino 10125, Italy; Department of Life Sciences and Systems Biology, University of Torino, Torino 10125, Italy; Biological and Environmental Sciences and Engineering Division, Center for Desert Agriculture (CDA), King Abdullah University of Science and Technology (KAUST), The BioActives Lab, Thuwal, 23955-15 6900, Saudi Arabia; The Plant Science Program, Biological and Environmental Science and Engineering Division, King Abdullah University of Science and Technology (KAUST), Thuwal, Saudi Arabia; Plant Cell and Developmental Biology, Biological and Environmental Sciences and Engineering (BESE), King Abdullah University of Science and Technology (KAUST), Thuwal 23955-6900, Saudi Arabia; Biological and Environmental Sciences and Engineering Division, Center for Desert Agriculture (CDA), King Abdullah University of Science and Technology (KAUST), The BioActives Lab, Thuwal, 23955-15 6900, Saudi Arabia; Biological and Environmental Sciences and Engineering Division, Center for Desert Agriculture (CDA), King Abdullah University of Science and Technology (KAUST), The BioActives Lab, Thuwal, 23955-15 6900, Saudi Arabia; National Research Council, Institute for Sustainable Plant Protection, Turin 10135, Italy; Biological and Environmental Sciences and Engineering Division, Center for Desert Agriculture (CDA), King Abdullah University of Science and Technology (KAUST), The BioActives Lab, Thuwal, 23955-15 6900, Saudi Arabia; Biological and Environmental Sciences and Engineering Division, Center for Desert Agriculture (CDA), King Abdullah University of Science and Technology (KAUST), The BioActives Lab, Thuwal, 23955-15 6900, Saudi Arabia; Biological and Environmental Sciences and Engineering Division, Center for Desert Agriculture (CDA), King Abdullah University of Science and Technology (KAUST), The BioActives Lab, Thuwal, 23955-15 6900, Saudi Arabia; The Plant Science Program, Biological and Environmental Science and Engineering Division, King Abdullah University of Science and Technology (KAUST), Thuwal, Saudi Arabia; Plant Cell and Developmental Biology, Biological and Environmental Sciences and Engineering (BESE), King Abdullah University of Science and Technology (KAUST), Thuwal 23955-6900, Saudi Arabia; Department of Life Sciences and Systems Biology, University of Torino, Torino 10125, Italy; Biological and Environmental Sciences and Engineering Division, Center for Desert Agriculture (CDA), King Abdullah University of Science and Technology (KAUST), The BioActives Lab, Thuwal, 23955-15 6900, Saudi Arabia; The Plant Science Program, Biological and Environmental Science and Engineering Division, King Abdullah University of Science and Technology (KAUST), Thuwal, Saudi Arabia

## Abstract

Carotenoid cleavage, catalyzed by CAROTENOID CLEAVAGE DIOXYGENASEs (CCDs), provides signaling molecules and precursors of plant hormones. Recently, we showed that zaxinone, a apocarotenoid metabolite formed by the CCD ZAXINONE SYNTHASE (ZAS), is a growth regulator required for normal rice (*Oryza sativa*) growth and development. The rice genome encodes three *OsZAS* homologs, called here *OsZAS1b*, *OsZAS1c*, and *OsZAS2*, with unknown functions. Here, we investigated the enzymatic activity, expression pattern, and subcellular localization of OsZAS2 and generated and characterized loss-of-function CRISPR/Cas9 (clustered regularly interspaced short palindromic repeats and associated protein 9)-*Oszas2* mutants. We show that OsZAS2 formed zaxinone in vitro. OsZAS2 was predominantly localized in plastids and mainly expressed under phosphate starvation. Moreover, *OsZAS2* expression increased during mycorrhization, specifically in arbuscule-containing cells. *Oszas2* mutants contained lower zaxinone content in roots and exhibited reduced root and shoot biomass, fewer tillers, and higher strigolactone (SL) levels. Exogenous zaxinone application repressed SL biosynthesis and partially rescued the growth retardation of the *Oszas2* mutant. Consistent with the *OsZAS2* expression pattern, *Oszas2* mutants displayed a lower frequency of arbuscular mycorrhizal colonization. In conclusion, *OsZAS2* is a zaxinone-forming enzyme that, similar to the previously reported OsZAS, determines rice growth, architecture, and SL content, and is required for optimal mycorrhization.

## Teaser

OsZAS2 is a root- and arbusculated cell-localized zaxinone-forming enzyme required for proper rice growth and normal mycorrhization and SL homeostasis.

## Introduction

Carotenoids are tetraterpene (C_40_) pigments consisting of long hydrocarbon chains with a conjugated double-bond system. In plants, carotenoids serve as a crucial component of photosynthesis, colorants, and antioxidants (Bouvier et al., 2003; Fraser and Bramley, 2004; Ballottari et al., 2014; Nisar et al., 2015; Hashimoto et al., 2016; Rodriguez et al., 2018; Zheng et al., 2020). In addition, the breakdown of carotenoids gives rise to a diverse group of metabolites called apocarotenoids, which includes pigments, scents, signaling molecules, growth regulators, and the precursors of the phytohormones strigolactone (SL) and abscisic acid (ABA) (Felemban et al., 2019; Moreno et al., 2021; Wang et al, 2021a; Liang et al., 2021; Zheng et al., 2021). ABA is the most-studied plant apocarotenoid hormone and a key player in plant response to abiotic and biotic stress (Peleg and Blumwald, 2011), regulation of seed maturation, dormancy, and shoot and root growth (Nambara and Marion-Poll, 2005; Moreno et al., 2021). SLs regulate a series of developmental processes. They are best known for inhibiting shoot branching/tillering, regulating root architecture, secondary growth, and senescence, and for their contribution to biotic and abiotic stress responses (Gomez-Roldan et al., 2008; Umehara et al., 2008; Ha et al., 2014; Al-Babili and Bouwmeester, 2015; Decker et al., 2017; Waters et al., 2017; Jia et al., 2019). However, SLs were originally discovered as host root-released germination stimulants for seeds of root parasitic weeds (Xie et al., 2010). Later on, they were identified as the plant-released hyphal branching factor for arbuscular mycorrhizal (AM) fungi, which paved the way for establishing plant–AM symbiosis (Akiyama et al., 2005). AM fungi symbiotic association provides the host plant with minerals, mainly phosphorus (P) and nitrogen (N), and the AM fungi with carbohydrates and lipids (Wang et al., 2017). AM symbiosis is widely distributed and formed by most of the land plants, mirroring its importance for their growth and survival (Gutjahr and Parniske, 2013; Wang et al., 2017; Fiorilli et al., 2019).

Recent studies unraveled the presence of several apocarotenoid signaling molecules, such as anchorene, iso-anchorene, β-cyclocitral, and zaxinone. Anchorene is a carotenoid-derived dialdehyde responsible for anchor root formation in Arabidopsis (*Arabidopsis thaliana*) (Jia et al., 2019), while its structural isomer iso-anchorene inhibits Arabidopsis root growth (Jia et al., 2021). β-Cyclocitral regulates root growth and is a retrograde signaling molecule that mediates singlet oxygen response and improves the high light tolerance by modulating the expression of oxidative stress-responsive genes (Ramel et al., 2012; Dickinson et al., 2019). Zaxinone is a regulatory metabolite, which is required for normal rice (*Oryza sativa*) growth and development and negatively regulates SL biosynthesis (Wang et al., 2019, 2020). Multi-omics study revealed that zaxinone also modulates cytokinin homeostasis and that its growth-promoting effect is likely caused by increased sugar metabolism in rice roots (Wang et al., 2021a, 2021b). However, exogenous application of zaxinone to Arabidopsis simultaneously increased both SL and ABA content (Ablazov et al., 2020), suggesting that it might act as a stress signal in Arabidopsis (Ablazov et al., 2020).

Apocarotenoid production in plants is mediated by carotenoid cleavage dioxygenases (CCDs), which cleave double bonds in carotenoid backbones and exhibit different substrate and cleavage site specificities (Moise et al., 2014; Dhar et al., 2020). The diversity of CCDs gives rise to a wide spectrum of apocarotenoids with unique features and functions (Giuliano et al., 2003; Auldridge et al., 2006; Ahrazem et al., 2016). Based on phylogenetic analysis and enzymatic activity, plant CCDs build six subfamilies; NINE-CIS-EPOXY CAROTENOID DIOXYGENASEs (NCEDs), CCD1, CCD4, CCD7, CCD8, and ZAXINONE SYNTHASE (ZAS) (Wang et al., 2019). NCEDs are involved in ABA biosynthesis and catalyze the cleavage of the C11, C12 (or C11′, C12′) double bond in 9-cis-epoxy carotenoids to yield the ABA precursor xanthoxin (Schwartz et al., 1997; Chernys and Zeevart, 2000). In contrast to other CCD types investigated so far, CCD1 enzymes are localized in the cytosol. Moreover, they are characterized by wide substrate and regio-specificity, cleaving many carotenoid and apocarotenoid substrates and producing dialdehyde products and volatiles that contribute to the flavor and aroma in many plants (Vogel et al., 2008; Ilg et al., 2009, 2014). CCD4 enzymes cleave the C9–C10 or C9′–C10′ double bond in bicyclic carotenoids, giving rise to C_13_ volatiles and C_27_-apocarotenoids (Bruno et al., 2015, 2016). CCD7 and CCD8 act sequentially on 9-cis-β-carotene to produce carlactone, the central intermediate of SL biosynthesis, via the intermediate 9-cis-β-apo-10′-carotenal formed by CCD7 along with the volatile β-ionone (Alder et al., 2012; Bruno et al., 2014). Carlactone is further modified by cytochrome P450s (711 clades), such as the Arabidopsis MORE AXILLARY GROWTH1 or the rice carlactone oxidase, leading to the formation of canonical and noncanonical SLs (Abe et al., 2014; Zhang et al., 2014; Jia et al., 2018; Ito et al., 2022).

ZAS is a recently discovered member of the CCD family, which cleaves the apocarotenoid apo-10-zeaxanthinal (C_27_) at the C13, C14 double bond, forming the C_18_-apocarotenoid zaxinone (Wang et al., 2019). Zaxinone is a growth-promoting metabolite required for normal rice growth and a negative regulator of SL biosynthesis and release (Wang et al., 2019, 2020). A rice loss-of-function *zas* mutant showed reduced root zaxinone level, retarded growth, that is, lower root and shoot biomass, tiller number, and higher SL content (Wang et al., 2019). Confirming its biological function, the exogenous application of zaxinone restored several phenotypes of the *zas* mutant (Wang et al., 2019). Though all other CCD subfamilies are conserved, nonmycorrhizal species, such as *A. thaliana* and other members of the *Brassicales*, lack *ZAS* orthologs, indicating a role of *ZAS* in AM symbiosis (Fiorilli et al., 2019; Wang et al., 2019). Indeed, the *zas* mutant displayed a lower level of AM colonization compared to the wild-type (WT; Wang et al., 2019).

The rice genome contains three *OsZAS* homologs, previously called *OsZAS-L1* (renamed here to *OsZAS1b)*, *OsZAS-L2* (renamed here to *OsZAS1c)*, and *OsZAS-L3* (renamed here to *OsZAS2*) with unknown function (Wang et al., 2019). In this study, we investigated the biological function of OsZAS2 by studying its enzymatic activity and by generating and characterizing the corresponding mutant and GUS reporter lines. Obtained data suggest that OsZAS2 is a nonredundant, root- and arbusculated cell-localized zaxinone-forming enzyme required for proper growth and normal SL homeostasis and mycorrhization level.

## Results

### OsZAS2 represents a separate clade in the ZAS CCD subfamily

**Figure 1:**
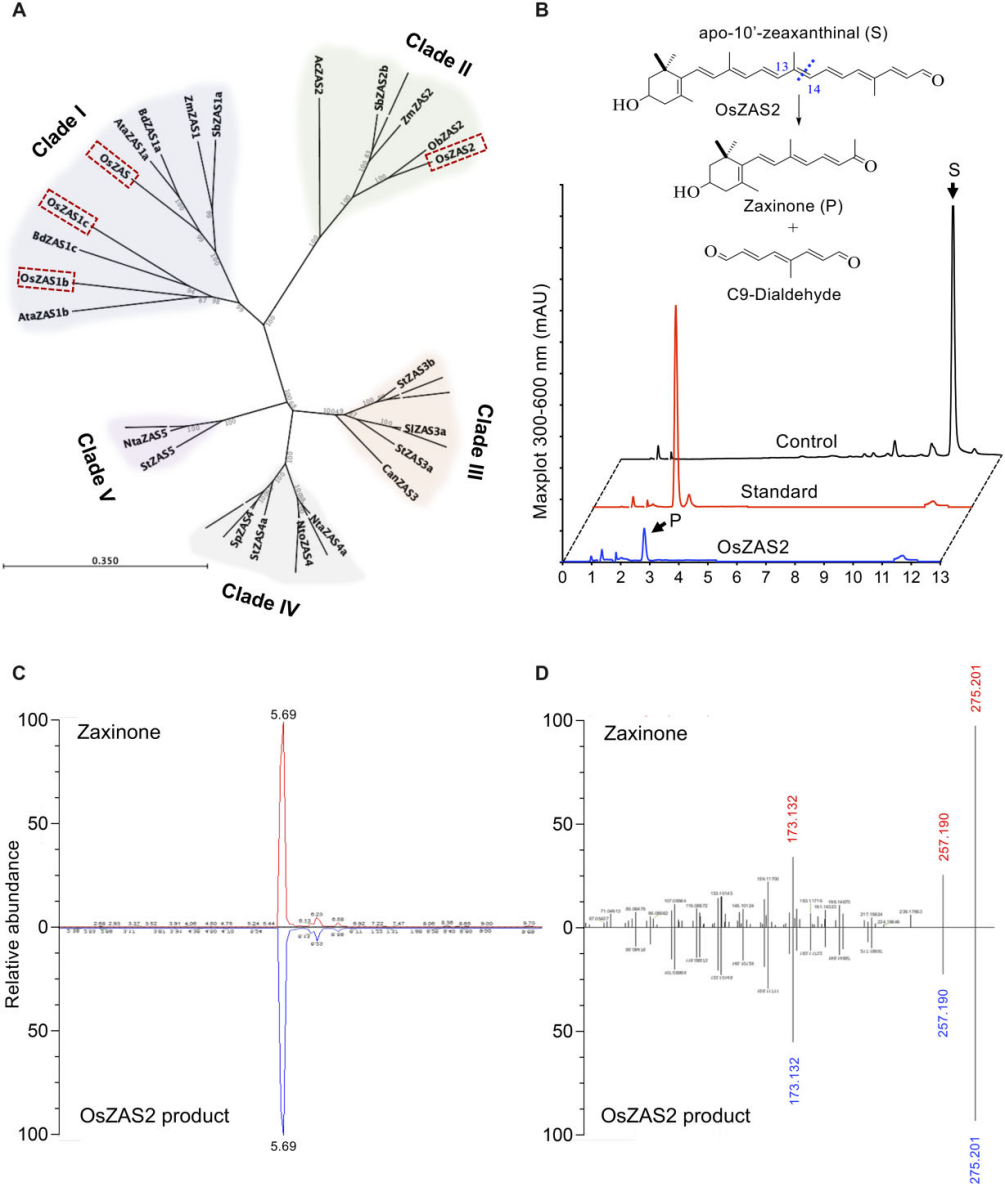
Phylogenetic analysis of ZAS enzymes and analysis of OsZAS2 enzymatic activity. A, Phylogenetic tree analysis of ZAS orthologs from selected monocot and dicot plants, showing bootstrap values on nodes of each cluster. Dashed rectangles represent rice ZAS members. The scale bar represents the number of amino acid change per site. B, HPLC chromatogram of in vitro incubation of OsZAS2 with apo-10′-zeaxanthinal (I) yielded zaxinone (II) and a presumed C_9_-dialdehyde. The maximum absorbance (mAU) peak for substrate and product is shown at 347 and 450 nm (mAU), respectively. The representation of zaxinone production in the Figure B adapted from Wang et al. (2019), which is permitted to adaptation under a Creative Commons Attribution 4.0 International License. C, Verification of OsZAS2 in vitro product, based on retention time, (D) accurate mass and MS/MS pattern and in comparison to zaxinone standard.

To clarify the phylogenetic relationship of *OsZASs* (*OsZAS*, *OsZAS-L1*, *OsZAS-L2*, and *OsZAS-L3*) with other plant *ZAS* genes, we first constructed a phylogenetic tree, using ZAS sequences from selected monocot and dicot species (Supplemental Table S2; Supplemental File S1). This analysis divided the ZAS proteins into five clades (I–V) (Figure 1A). Three OsZAS enzymes, including OsZAS (Os09g0321200), OsZAS-L1 (Os08g0369800), and OsZAS-L2 (Os08g0371608) grouped in clade I, while OsZAS-L3 (Os06g0162550) clustered in clade II. Based on this analysis we renamed OsZAS-L1, OsZAS-L2, and OsZAS-L3 to OsZAS1b, OsZAS1c, and OsZAS2, respectively (Figure 1A). The clades III, IV, and V contain only enzymes from dicot species, which we called ZAS3, ZAS4, and ZAS5 (Supplemental Table S3).

### OsZAS2 is a zaxinone-forming enzyme

Next, we investigated the enzymatic activity of OsZAS2: we expressed OsZAS2 fused to maltose-binding protein (MBP) in *Escherichia coli* cells and incubated the soluble fraction of these cells with different apocarotenoids, that is, β-apo-10′-(C_27_), 9-cis-β-apo-10′-(C_27_), β-apo-12′-(C_25_), and apo-8′-zeaxanthinal (3-OH-β-apo-8′-carotenal, C_30_) (Supplemental Figure S1). In addition, we incubated the MBP–OsZAS2 fusion with carotenoids, that is, β-carotene, zeaxanthin, and lutein (Supplemental Figure S1). Finally, we performed in vivo activity test by expressing a thioredoxin–OsZAS2 fusion in β-carotene, zeaxanthin, and lycopene-accumulating *E. coli* cells. In all these assays, we only detected a conversion of apo-10′-zeaxanthinal (C_27_) that was cleaved by OsZAS2 at the C13, C14 double bond, yielding zaxinone (3-OH-β-apo-13-carotenone, C_18_) and a predicted C_9_-dialdehyde (Figure 1B). We confirmed the identity of zaxinone by UHPLC (Ultra High Performance Liquid Chromatography) and LC–MS (Liquid chromatography–mass spectrometry) analysis, using a synthetic standard (Figure 1, C and D).

### OsZAS2 is predominantly localized in the plastid

**Figure 2:**
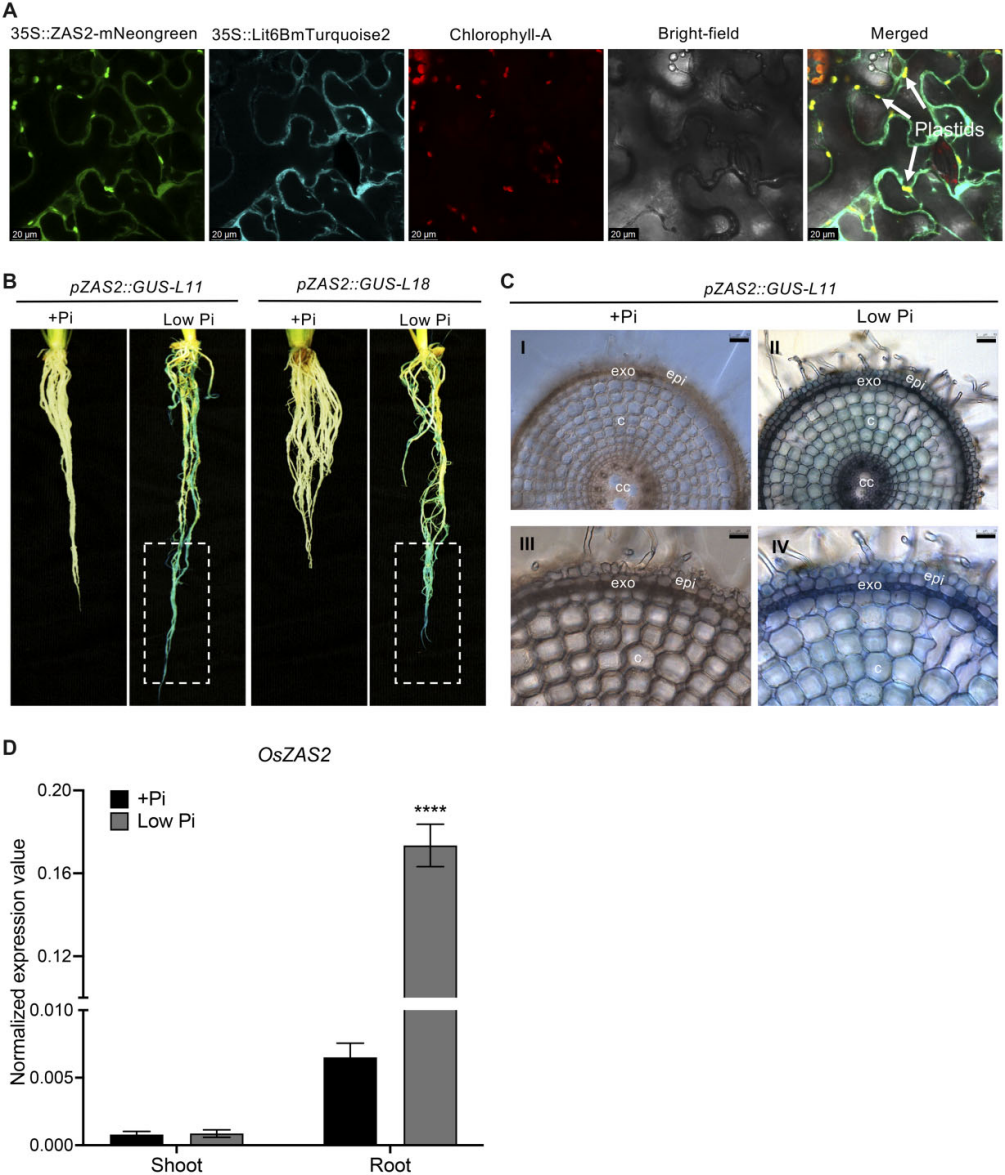
*OsZAS2* subcellular localization and expression pattern. A, Subcellular localization of OsZAS2 transiently expressed in *N. benthamiana* epidermis leave tissue. B, GUS staining of roots of two independent *pZAS2:GUS* reporter lines (*pZAS2::GUS-L11, pZAS2::GUS-L18*) under normal (+Pi) and low Pi conditions. Dash rectangle emphasizes the root tip. C, Cross-section of *pZAS2:GUS11* line primary root was examined with two different resolutions under microscope: in the first resolution (parts I and II, bars correspond to 50 μm) all types of tissues were observed while the second resolution of the same samples (parts III and IV, bars correspond to 25 μm) showed a close view of epidermal and cortex cells. Exo, exoderms; epi, epidermis; c, cortex; cc, central cylinder. D, Normalized expression value of OsZAS2 under normal (+Pi) and low Pi conditions in root and shoot tissue of 21-day-old rice plants. Values in (D) are means ± sd (*n* = 4). Student’s *t* test used for the statistical analysis (****P ≤ 0.0001).

The ChloroP Server program (Emanuelsson et al., 1999) predicts the presence of a plastid transit peptide in the OsZAS2, indicating a plastid localization of this enzyme. To confirm this prediction, we transiently expressed *OsZAS2* cDNA fused with the sequence encoding mNeonGreen fluorescence protein under the control of the *35S* promoter (*35S:OsZAS2:mNeonGreen*) in *Nicotiana benthamiana* leaves epidermal cells, alone or together with the gene encoding the plasma membrane specific Turquoise2 marker protein (*35S::Lit6BmTurquoise2*) (Cutler et al., 2000). As shown in Figure 2A, the green fluorescent signal of the OsZAS2 fusion clearly overlapped with the red autofluorescence of chlorophyll A. However, it also showed co-localization with the 35S::Lit6B Turquoise marker of the plasma membrane. Overall, we observed a stronger green fluorescent signal of the OsZAS2 fusion in plastids than in plasma membranes, supporting a plastid localization of this enzyme.

### OsZAS2 is expressed in roots and induced under low Pi

To determine the expression pattern of *OsZAS2*, we generated the *GUS* reporter lines *pOsZAS2::GUS11* and *pZAS2::GUS18* by fusing a 1.2-kb upstream *OsZAS2* fragment to *GUS* and transforming the resulting *pZAS2-MDC162* plasmid into rice. The staining of the two reporter lines showed that *OsZAS2* expression significantly increased under low Pi conditions, while the GUS signal was not detectable at all under normal conditions (Figure 2B). Moreover, the GUS signal increased substantially toward the tip of the primary and crown roots. The RT-qPCR (Reverse transcription-quantitative polymerase chain reaction) analysis also showed that the *OsZAS2* transcript level increased about 20-fold under low Pi compared to normal (+Pi) conditions in roots (Figure 2D). We further investigated OsZAS2 localization at the cellular level using cross-sectioning. Using a confocal microscope, we detected a strong GUS signal in the exodermis of primary roots, while the cortex, epidermis, and other root tissues showed only mild signals (Figure 2C).

### CRISPR/Cas9-generated *Oszas2* mutant lines show severe growth defects

**Figure 3:**
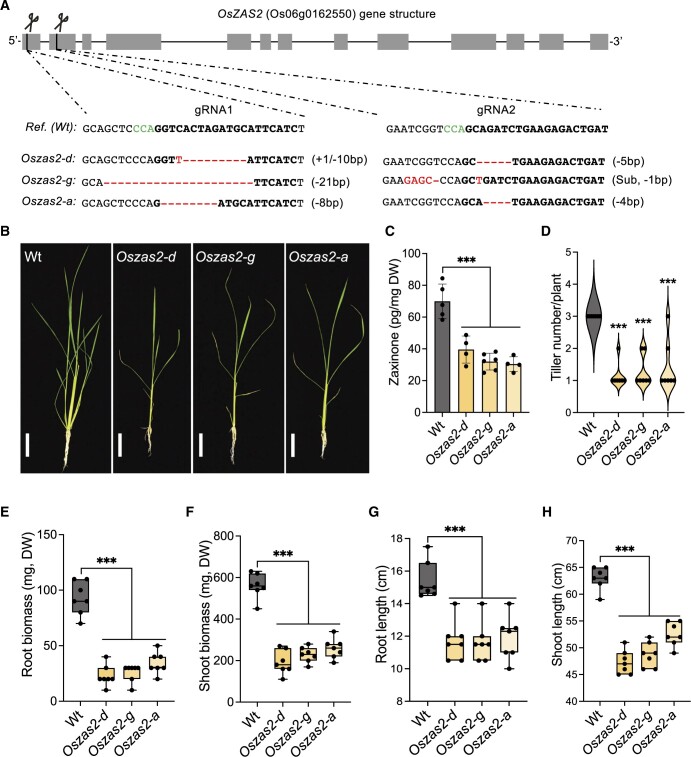
Characterization of CRISPR/Cas9-mediated *Oszas2* mutant lines at the seedling stages. A, Schematic representation of three individual mutations of *OsZAS2* gene generated by CRISPR–Cas9. B, The seedling phenotype of WT (DJ) and three independent *Oszas2* mutants. The scale bar in the pictures represents 7.5 cm. C, Quantification of zaxinone content in WT and *Oszas2* mutants roots. D–H, Root biomass (D), shoot biomass (E), root length (F), shoot length (G), and tiller number (H) of the WT and *Oszas2* mutants are shown in (B). Boxes in boxplots represent the median, first and third quartile. The minimum and maximum values are shown with the length of the whiskers. Dots represent the biological replicates. Values in (C–H) are means ± sd (*n* ≥ 4). Student’s *t* test used for the statistical analysis (***P ≤ 0.001).

**Figure 4:**
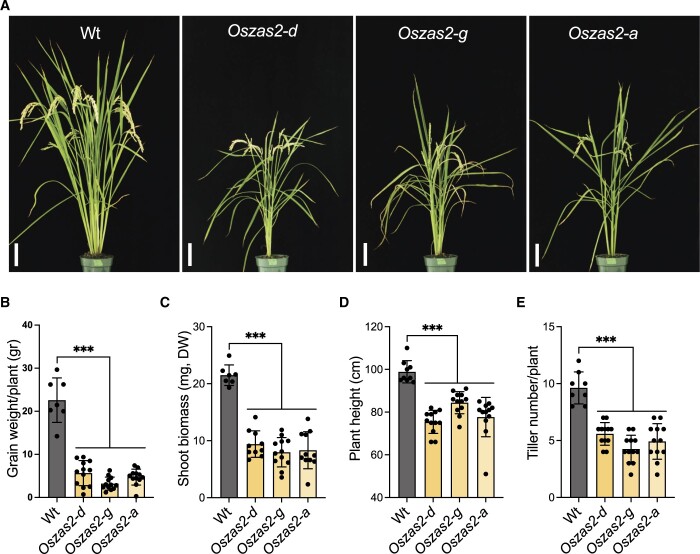
Characterization of Oszas2 mutant lines at the maturing stage. A, The picture of the 3-month-old WT and *Oszas2* mutants grown in the greenhouse. The scale bar in the pictures represents 7.5 cm. B–E, Grain weight per plant (B), shoot biomass (C), plant height (D), and tiller number (E) of the WT and *Oszas2* mutants represented in (A). Values in (B–E) are means ± sd (*n* ≥ 7). Student’s *t* test was applied for the statistical analysis (***P ≤ 0.001).

Next, we generated *Oszas2* mutant knockout lines in the Dongjin (DJ) variety by employing CRISPR/Cas9. For this purpose, we used two guide RNAs, gRNA1 and gRNA2, which target coding sequence in exons 1 and 2, respectively (Figure 3A). We identified three independent *Oszas2* mutants, *zas2-d*, *zas2-g*, and *zas2-a*, with mutations in the first and second exon (Figure 3A), which resulted in premature stop codons (Supplemental Figure S5). To validate the function of OsZAS2 as a zaxinone synthesizing enzyme in planta, we quantified zaxinone in roots of the three mutant lines grown hydroponically under normal and low Pi supply. Compared to WT, zaxinone content was reduced up to 45% in *Oszas2* mutant lines under normal conditions (Figure 3C), but was unchanged under low Pi conditions (Supplemental Figure S4A). Next, we grew the mutant lines in soil and characterized them at the seedling and mature stage. At seedling stage, *Oszas2* mutants displayed shorter roots and shoots and a striking reduction in root and shoot biomass (Figure 3, B and E–H). Moreover, they produced one tiller on average, while the WT developed three (Figure 3D). The low-tillering and reduced shoot biomass phenotypes remained pronounced after growing the mutants for 3 months in GH and caused a significant reduction in grain weight per plant, compared to the corresponding WT (Figure 4, A–E).

### *OsZAS2* is involved in AM colonization

**Figure 5:**
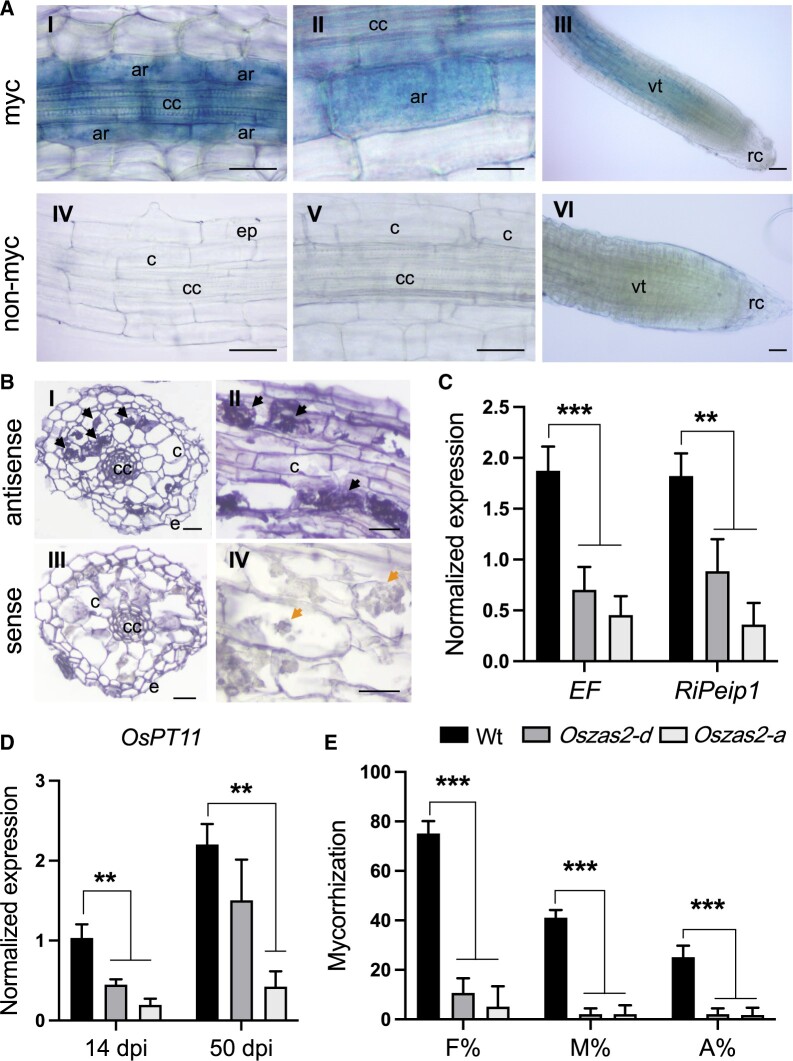
*OsZAS2* is required for AM establishment. A, GUS staining of roots of pZAS2:GUS-L18 reporter line inoculated (I, II, and III) for 35 days with *F. mosseae* and noninoculated (IV, V, and VI). B, Localization of *OsZAS2* mRNA in sections from differentiated regions of inoculated roots by cold in situ hybridization. Sections of mycorrhizal roots treated with *OsZAS2* antisense probe are shown in parts I and II; arrows highlight the strong chromogenic signal, which mirrors the presence of the *OsZAS2* transcript in arbuscule-containing cells. Sections of mycorrhizal roots treated with the *OsZAS2* sense probe are shown in parts III and IV; arrows in part IV indicate arbusculated cells that are not labeled. C, Relative expression of fungal genes; *RiEF* and *RiPeip1* in WT and *Oszas2* mutants at 50 dpi. D, Relative expression of *OsPT11* at 14 and 40 dpi in WT and *Oszas2* mutants. E, Frequency of mycorrhizal colonization (F%), the intensity of colonization (M%), and a total number of arbuscules (A%) in WT and *Oszas2* mutants at 50 dpi. cc, central cylinder; c, noncolonized cortical cells; e, epidermal cells; vt, vascular tissue; ar, arbuscule containing cells; rc, root cap. Bars (A and B) correspond to 50 µm. Values in (C–E) are means ± sd (*n* ≥ 3). Student’s *t* test was applied for the statistical analysis (**P ≤ 0.01; ***P ≤ 0.001).

The expression analysis of *OsZAS2* in whole roots at early and late stages of AM colonization revealed an upregulation at 21 days postinoculation (dpi) (Supplemental Figure S2A), when arbuscules are present, as witnessed by the abundance of the AM-inducible plant marker *OsPT11* transcript (Paszkowski et al., 2002; Supplemental Figure S2B). To obtain deeper insights into the spatial expression of *OsZAS2* during mycorrhization, we inoculated *pZAS2::GUS* reporter lines with AM fungi and monitored the GUS signal. Interestingly, we detected GUS activity only in arbusculated cells (Figure 5A; Supplemental Figure S2C), and did not observe any signal in any other root cells, including cortical cells and cells crossed by fungal hyphae. We further validated this observation by using in situ hybridization assays on mycorrhizal roots of WT plants. *OsZAS2* mRNA exclusively accumulated in cells with fully developed arbuscules, in which we detected a strong chromogenic signal (Figure 5B). We did not observe any signal in noncolonized cells or upon using the *OsZAS2* sense probe (Figure 5B).

To determine the impact of *OsZAS2* on AM symbiosis, we inoculated the *Oszas2* mutant lines with AM fungi and assessed the colonization level by morphological analysis and by monitoring the transcript abundance of *OsPT11* at two time points (14 and 50 dpi) and of the fungal genes *RiEF* and *RiPeip1* (Fiorilli et al., 2016) at 50 dpi. Both mutant lines showed a lower frequency of mycorrhizal colonization (F%), intensity of colonization (M%), and total number of arbuscules (A%), compared to WT plants (Figure 5E). Molecular analysis confirmed these results: the expression level of fungal and plant genes was significantly lower in the mutant lines (Figure 5, C and D).

### SL biosynthesis increased in *Oszas2* mutants

**Figure 6:**
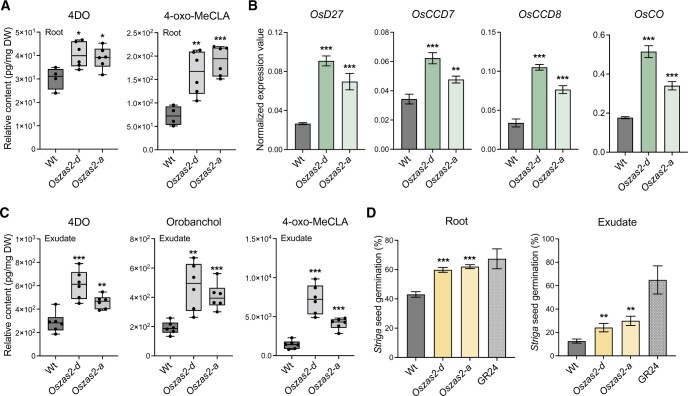
SL biosynthesis increased in *Oszas2* mutants. A, Relative quantification of 4DO and 4-oxo-MeCLA in root tissue of *Oszas2* mutants. B, Normalized expression value of SL biosynthetic genes in *Oszas2* mutants. C, Relative quantification of canonical (4DO, Orobanchol) and noncanonical (4-oxo-MeCLA) SL in root exudate of *Oszas2* mutants. D, *Striga* seed germination assay conducted with root exudate and tissue of *Oszas2* mutants. For both root exudate and tissue bioassay, 1 μM of GR24 was used as control, which showed about 65% and 67% of *Striga* seed germination, respectively. Boxes in boxplots in (A) and (C) represent the median, first and third quartile. The minimum and maximum values are showed with the length of the whiskers. Dots represent the biological replicates. Values in (A–D) are means ± sd (*n* ≥ 4) and student’s *t* test was applied for the statistical analysis (*P ≤ 0.05; **P ≤ 0.01; ***P ≤ 0.001).

The low-tillering phenotype of *Oszas2* mutants (Figures 3, D and 4, E) indicated that they may have higher SL content. To test this hypothesis, we profiled their SLs in both roots and root exudates under low Pi conditions. We also analyzed the expression level of SL biosynthetic genes in roots. In roots, the contents of a noncanonical SL, a tentative 4-oxo-methylcarlactonate (4-oxo-MeCLA) (Yoneyama et al., 2018; Ito et al., 2022), and of the canonical SL 4-deoxyorobanchol (4DO) were significantly increased in *Oszas2* lines compared to WT (Figure 6A). As shown in Figure 6B, we also observed an increase in the transcript level of SL biosynthetic genes, that is, *D27*, *CCD7*, *CCD8*, and *OsCO*. To confirm the increase in SLs, we conducted *a Striga* seed germination assay with root extracts. Extracts of *Oszas2* roots showed a significantly higher germination rate (around 60%), compared to those of WT (around 42%, Figure 6D). In root exudates, both canonical (4DO, orobanchol) and noncanonical (4-oxo-MeCLA) SLs were significantly increased in *Oszas2* mutants, compared to WT (Figure 6C). Here again, we performed a *Striga* seed germination assay, in which *Oszas2* mutant lines displayed about15% higher activity compared to WT (Figure 6D), which confirms the LC–MS quantification.

### Exogenous zaxinone application repressed SL biosynthesis in *Oszas2* mutant

**Figure 7:**
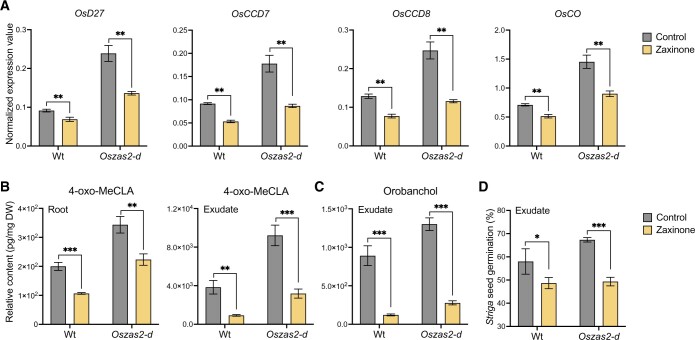
Zaxinone treatment reduced SL biosynthesis in *Oszas2* mutant. A, SL biosynthetic genes; *OsD27*, *OsCCD7*, *OsCCD8*, and *OsCO* expression in WT and *Oszas2* mutant upon zaxinone (5 μM) treatment. B, Relative content of 4-oxo-MeCLA after zaxinone (5 μM) treatment in root tissue and exudate of WT and *Oszas2* mutant. C, Relative content of Orobanchol after zaxinone (5 μM) treatment in root exudate of WT and *Oszas2* mutant. D, *Striga* seed germination assay with exudate of WT and *Oszas2* mutant upon zaxinone (5 μM) treatment. About 1 μM of GR24 was used as control, which showed about 63% of *Striga* seed germination. Values in (A–D) are means ± sd (*n* ≥ 4). Student’s *t* test was applied for the statistical analysis (*P ≤ 0.05; **P ≤ 0.01; ***P ≤ 0.001).

Next, we treated 3 weeks old, hydroponically grown *Oszas2* seedlings (grown 1 week in Hoagland agar and 2 weeks in low Pi) with 5 μM zaxinone for 6 h. As shown in Figure 7A, zaxinone treatment repressed transcript levels of the SL biosynthetic genes *D27*, *CCD7*, *CCD8*, and *OsCO* in *Oszas2-d* back to the WT level. Furthermore and as shown before (Wang et al., 2019, 2020), exogenous zaxinone application decreased the transcript level of SL biosynthetic genes in WT as well (Figure 7A). Zaxinone application also decreased the content of the noncanonical SL 4-oxo-MeCLA in roots and root exudates of *Oszas2-d* and WT (Figure 7B). In addition, it caused a reduction in the level of the canonical SL orobanchol in root exudates of both *Oszas2-d* and WT (Figure 7C). Surprisingly, 4DO content was slightly increased upon zaxinone treatment in root tissues of both *Oszas2-d* and WT while it was not affected in root exudates (Supplemental Figure S3). Moreover, root exudates of both *Oszas2* and WT plants showed, upon zaxinone treatment, decreased Striga seed germination (Figure 7D).

### Zaxinone treatment partially rescued the growth defects of *Oszas2* mutant

**Figure 8:**
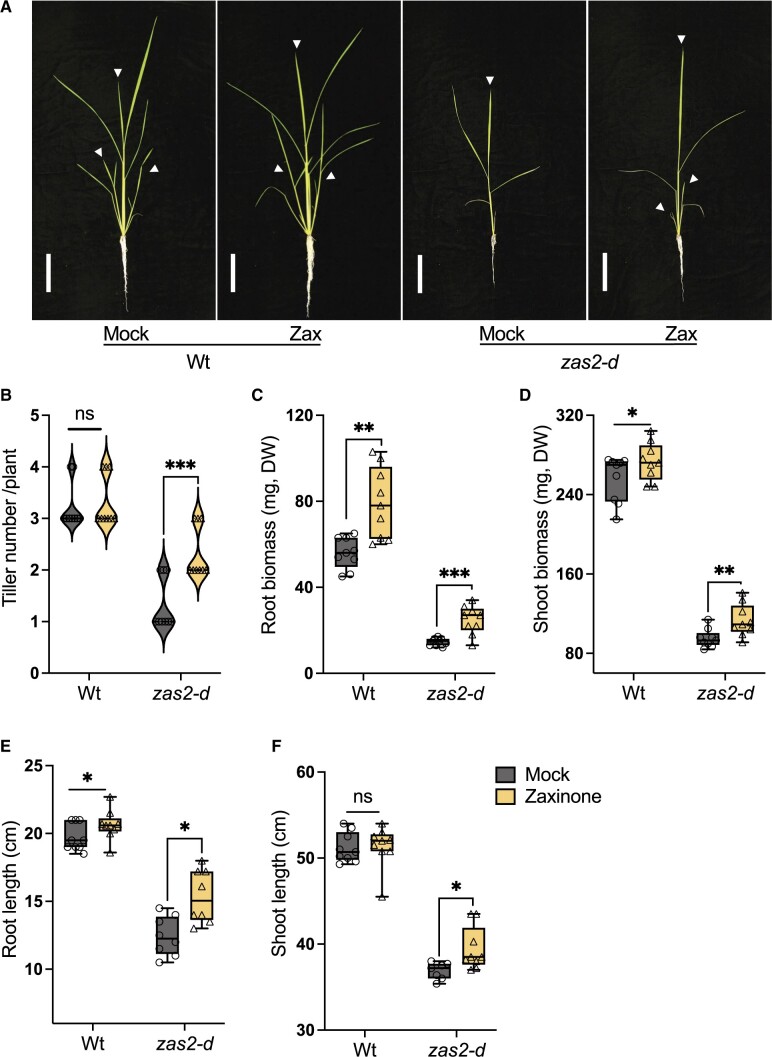
Exogenous zaxinone application rescued the growth defects of *Oszas2* mutant. A, The images of the WT (DJ) and *Oszas2-d* mutant grown for 2 weeks in soil supplemented with 10 μM of zaxinone and tap water (0.01% [v/v] acetone) as mock. The white bar represents 10 cm of scale. The white arrows represent main tillers. B–F, Tiller number (B), root biomass (C), shoot biomass (D), root length (E), and shoot length (F) of the WT and Oszas2 mutants are shown in (A). Boxes in boxplots represent the median, first and third quartile. The minimum and maximum values are showed with the length of the whiskers. Dots represent the biological replicates. Values in (B–F) are means ± sd (*n* ≥ 7). Student’s *t* test used for the statistical analysis (*P ≤ 0.05; **P ≤ 0.01; ***P ≤ 0.001; ns, not significant).

Next, we supplied *Oszas2* seedlings, grown in soil, with exogenous zaxinone at a concentration of 10 μM. After 2 weeks, we observed an increase in the tiller number of *Oszas2-d* mutant, but not of that of the WT (Figure 8, A and B). Moreover, zaxinone treatment significantly increased root and shoot biomass and shoot length of *Oszas2-d* (Figure 8, C–F). We also observed an increase in root length and root and shoot biomass of treated WT plants upon zaxinone treatment, while their shoot length remained unaffected (Figure 8, C, D, and F).

## Discussion

The identification of a zaxinone synthase, the rice ZAS, revealed the presence of a widely distributed plant CCD subfamily (Wang et al., 2019). Functional studies and characterization of a corresponding T-DNA insertion mutant demonstrated the importance of *ZAS* for plant growth, interaction with AM fungi, and SL homeostasis. Furthermore, exogenous treatment with zaxinone revealed the growth-promoting effect of this apocarotenoid and its impact on hormone homeostasis and sugar metabolism, indicating that it may be a candidate for a novel plant hormone (Wang et al., 2019, 2021a, 2021b). Rice contains three *OsZAS* homologs (Wang et al., 2019), called here *ZAS1b*, *ZAS1c*, and *ZAS2*, with unknown functions. The severe phenotypes of *zas* mutant indicate the importance of this gene and suggest that its activity cannot be compensated by that of its homolog(s), and that the latter may exert different function(s). Therefore, we were interested in investigating the biological function of these enzymes.

Phylogenetic analysis placed OsZAS2 in a clade different from that of ZAS, ZAS1b, and 1c, indicating a different biological role and maybe enzymatic activity (Figure 1A). Therefore, we focused in this study on OsZAS2. However, the enzymatic studies performed here demonstrate that OsZAS2 catalyzes the same reaction as ZAS, that is, it converts apo-10′-zeaxanthinal into zaxinone (Figure 1B). Indeed, the enzyme did not convert other substrates tested, for example, β-carotene, zeaxanthin, or different apocarotenoids, or produced other products, pointing to zaxinone formation as its enzymatic function. The same activity was reported for OsZAS. However, OsZAS cleaved, in addition to apo-10′-zeaxanthinal, apo-12′- and apo-8′-zeaxanthinal, but with lower activity (Wang et al., 2019). This difference might be caused by a wider substrate specificity. However, it is also possible that the heterologously expressed OsZAS is generally more active than ZAS2.

To explore the biological function of OsZAS2 in planta, we generated *Oszas2* knockout lines using the CRISPR/Cas9 technology. Disrupting *OsZAS2* led to around 40% decrease in roots zaxinone content. This decrease supports the in vitro enzymatic activity of OsZAS2 and suggests that OsZAS2 is an enzyme, besides OsZAS, responsible for zaxinone biosynthesis in rice. *Oszas2* mutants still contained a substantial amount of zaxinone in roots. This could be due to the activity of OsZAS, which might compensate for the zaxinone production in rice roots. Nevertheless, zaxinone is common at higher levels in green tissues than in roots and is also present in plant species, such as Arabidopsis, which lacks *ZAS* genes, indicating that it can be synthesized via alternative route(s) independent of ZAS enzymes (Mi et al., 2019; Wang et al., 2019; Ablazov et al., 2020). However, the phenotypes observed with *Oszas* and *Oszas2* mutants suggest the importance of these enzymes and indicate the zaxinone content in roots is crucial for normal growth and development. We are currently generating *Oszas*/*Oszas2* double mutants, which could give us a hint about the involvement of other route(s) in zaxinone biosynthesis.

Zaxinone is a negative regulator of SL biosynthesis in rice (Wang et al., 2019). Indeed, the *Oszas* mutant contained and released higher amounts of SLs (Wang et al., 2019) under Pi starvation, and this increase could be suppressed by exogenous zaxinone application. Based on its zaxinone-forming activity and the low-tillering phenotype of the *Oszas2* mutants, we assumed that the loss of OsZAS2 may also cause an increase in SL content. Therefore, we quantified the SL and zaxinone content in *Oszas2* mutants under low Pi conditions. Indeed, transcript levels of the SL biosynthetic genes *OsD27, OsCCD7, OsCCD8*, and *OsCO* transcript levels were upregulated in *Oszas2* compared to WT (Figure 6A). In parallel, both canonical and noncanonical SLs were significantly increased in roots and root exudates of *Oszas2* mutants (Figure 6, A and C), as confirmed by LC–MS analysis and by using the *Striga* seed germination bioassay. Interestingly, zaxinone content was not changed in *Oszas2* mutants under low Pi conditions (Supplemental Figure S4A), compared to the WT, albeit an increase in SL biosynthesis that is assumed to be caused by a decrease in zaxinone content. Since OsZAS is still functional in the *Oszas2* mutant, we hypothesized that it might compensate for the loss of *Oszas2* activity. In fact, *OsZAS* expression was upregulated, but not that of its homologs *OsZAS1b* and *OsZAS1c*, in *Oszas2* mutant under low Pi conditions (Supplemental Figure S4B). It might be speculated that changes in zaxinone content in certain root cells are crucial for regulating SL biosynthesis and that the increase in OsZAS activity may lead to a generally higher zaxinone content but cannot replace OsZAS2 in cells expressing this enzyme. Clarifying this point requires precise localization of both enzymes under low Pi conditions.

*Oszas2* mutants showed severe reduction in root and shoot biomass (Figure 3, E and F) and developed fewer tillers compared to WT (Figure 3D). The retarded growth of *Oszas2* mutants demonstrates that *OsZAS2* is necessary for normal rice growth and development. In general, the phenotypes of the *Oszas2* mutants, reduced zaxinone content, retarded growth, and reduced tiller number, are similar to that of the *Oszas* mutant under normal conditions (Wang et al., 2019). Hence, it can be concluded that rice requires both *ZAS* and *ZAS2* genes to keep the root zaxinone concentration at a certain level, as well as for normal rice growth and development under normal conditions.

We checked the expression pattern of *OsZAS2* at tissue and cellular level using RT-qPCR and promoter–GUS–reporter lines. Similar to *OsZAS* (Wang et al., 2019), *OsZAS2* is expressed in roots and induced upon Pi starvation. A robust upregulation of both *OZAS* and *OsZAS2* in rice roots in response to Pi starvation indicates their involvement in the plant’s response to Pi deficiency. Interestingly, analysis of the GUS reporter lines (*pOsZAS2::GUS11* and *pZAS2::GUS18*) demonstrated that the *OsZAS2* expression level was higher in root tips (Figure 2A). The cross-sectioning of the primary roots of the *pOsZAS2::GUS11* further showed that *OsZAS2* is highly expressed in exoderms. Here again, it would be very interesting to monitor *OsZAS* expression patterns at the cellular level to get insights into the function of *OsZAS* and *OsZAS2* and understand why both of them are important for proper rice growth.

In a previous work, we demonstrated that *zas* mutant showed a lower AM colonization level, compared to WT plants (Wang et al., 2019). Moreover, we revealed that the *ZAS* gene family is absent in genomes of nonmycorrhizal species, such as Arabidopsis (Fiorilli et al., 2019; Wang et al., 2019), suggesting a strong link between *ZAS* and AM symbiosis. To investigate the role of OsZAS2 in the different steps of AM colonization, we monitored its expression level during a time course experiment in mycorrhizal and nonmycorrhizal roots. Contrarily to *OsZAS* which was upregulated during both early and later stages, *OsZAS2* was only upregulated at the maximum of arbuscules formation (21 dpi), suggesting an involvement in arbuscules development/formation. This assumption is supported by in situ hybridization and using the *pZAS2::GUS* reporter lines: indeed both assays revealed that *OsZAS2* expression is localized in arbusculated cells. To further clarify the *OsZAS2* involvement during the AM symbiosis, we assessed the *Oszas2* mutant lines (*Oszas2-d* and *Oszas2-a*) colonization level at the morphological level and using molecular analyses. Although *Oszas2* mutants displayed in nonmycorrhizal condition a higher level of SLs in roots and root exudates, they showed a severe reduction of AM colonization level; however, no defects in arbuscules morphology were detected. A similar phenotype was also observed in the *Oszas* mutant (Wang et al., 2019). We recently demonstrated that the lower AM colonization rate of the *Oszas* mutant is due to SL deficiency at the early stage of the AM interaction (Votta et al., 2022): during this phase OsZAS activity is required to induce SL production possibly through the Dwarf14-Like (D14L) signaling pathway which was shown to regulate AM colonization in rice (Gutjahr et al., 2015). We can hypothesize that also OsZAS2 acts as a component of a regulatory network that involves SL and D14L pathways. Although further experiments are needed to prove this hypothesis, all the above data demonstrate that OsZAS2, in analogy to OsZAS, is required to reach a correct level of AM colonization.

We assume that the growth retardation of the *Oszas2* mutants is more likely due to decreased root zaxinone levels under normal conditions. Therefore, we applied zaxinone exogenously in to the *Oszas2-d* seedlings grown in soil. The exogenous application of zaxinone partially rescued the low-tillering, reduced root and shoot biomass, and shorter root and shoot length phenotypes of *Oszas2-d* mutant (Figure 8, C–F). This result indicates that a certain level of zaxinone is required to keep normal SL homeostasis and, hence, maintain normal tillering degree. Moreover, the effects of zaxinone treatment highlight again the importance of appropriate zaxinone concentrations of this apocarotenoid for regular growth and development of rice and support the idea of its function as a growth-promoting metabolite.

In conclusion, we revealed the function of *OsZAS2*, a member of the *CCD* gene family, which is crucial for normal rice growth and development. Besides OsZAS, OsZAS2 contributes to zaxinone production in rice roots and is a further determinant of SL content. Moreover, it is involved in regulating the levels of mycorrhizal colonization. Thus, manipulation of OsZAS2 expression level could be a tool to modulate rice architecture and improve AM symbiosis.

## Materials and methods

### Plant material and phenotyping

Rice seedlings (*O. sativa* L. cv DJ) were grown in a Biochamber under the following conditions: a 12-h photoperiod, 500 µmol photons m^−2^ s^−1^ and day/night temperature of 27/25°C. Briefly, rice seeds were surface sterilized in a 50% (v/v) household bleach for 15 min and rinsed 5 times with distilled water. Then, sterilized seeds were germinated in the dark for 2 days in the magenta boxes containing 50 mL of 0.4% (w/v) agarose half-strength Hoagland medium with pH 5.8 at 30°C. The pregerminated seeds were transferred to the biochamber and kept for 5 days.

For metabolite quantification, gene expression analysis, and *Striga* seed germination assay, 1-week-old rice seedlings were transferred into 50-mL black falcon tubes filled with half-strength modified Hoagland nutrient solution with adjusted pH to 5.8. The nutrient solution consisted of 5.6-mM NH_4_NO_3_, 0.8-mM MgSO_4_.7H_2_O, 0.8-mM K_2_SO_4_, 0.18-mM FeSO_4_.7H_2_O, 0.18-mM Na_2_EDTA.2H_2_O, 1.6-mM CaCl_2_.2H_2_O, 0.8-mM KNO_3_, 0.023-mM H_3_BO_3_, 0.0045-mM MnCl_2_.4H_2_O, 0.0003-mM CuSO_4_.5H_2_O, 0.0015-mM ZnCl_2_, 0.0001-mM Na_2_MoO_4_.2H_2_O, and 0.4-mM K_2_HPO_4_.2H_2_O. For normal conditions (+Pi), the 1-week-old seedlings were grown in the Hoagland nutrient solutions (+Pi) for another 2 weeks. For phosphate starvation, the seedlings were grown for 2 weeks in lower phosphate (4 µM, K_2_HPO4⋅2H_2_O) nutrient solution. The nutrient solution was replaced every 3 days. For zaxinone treatment, 3-week-old seedlings were treated with 5 µM of zaxinone for 6 h: tissues were collected and immediately frozen into liquid N_2_.

For phenotyping, 1-week-old *Oszas2* seedlings were transferred into pots filled with soil and grown in growth chamber under the above-mentioned conditions. Tap water was supplied when needed. After 18 days, roots were cleaned off from the soil. Then, the seedlings were photographed with a digital camera, and root and shoot lengths were measured. To analyze the dry weight (DW) of roots and shoots biomass, samples were kept for 3 days in a 65°C oven. For phenotyping of *Oszas2* mutants at the mature stage, 1-week-old seedlings were transferred into greenhouse and grown until the mature stage with a day/night temperature of 28°C/25°C. One-time tap water and a one-time half-strength nutrient solution were supplied when necessary. After 3 months, yield-related traits were recorded. This experiment was repeated twice.

For rescue experiments, 7-days-old seedlings were transferred into 1-L pots filled with soil and grown in Biochamber. Initially, for zaxinone treatment 200 mL of 10-µM zaxione solution (pH 5.8) was added per pot. The same volume of tap water containing 0.01% (v/v) acetone was used as control. Every 3 days, 50 mL of 10 µM of zaxione solution and an equivalent amount of water were added to the treatment and control groups, respectively. After 2 weeks of treatment, seedlings were phenotyped as above.

### Generation of transgenic lines

The CRISPR/Cas9 genome-editing technique was used to knock out *OsZAS2* (*Os06g0162550)* in *O. sativa ssp. japonica* variety DJ. The gRNA sequences were designed using the CRISPR-PLANT webserver (www.genome.arizona.edu/crispr/). Two different gRNA sites targeted *OsZAS2* in exon regions; at exon 1 which encodes (5′-GGTCACTAGATGCATTCATC-3′) and exon 2 which encodes (5′-GCAGATCTGAAGAGACTGAT-3′). The gRNA spacers were fused to a tRNA sequence as described by Xie et al. (2015) (Supplemental Figure S6) and synthesized from GENEWIZ (South Plainfield, NJ, USA). Then, the corresponding fragment was cloned into pRGEB32 (Kanamycin) using 5′-BsaI and 3′-BsaI sites. The *pRGEB32-OsZAS2* construct was further introduced into *Agrobacterium tumefaciens* strain *EHA105* competent cells via electroporation. To construct the *pOsZAS2::GUS* reporter plasmid, the 1.2-kb promoter region of *OsZAS2* was amplified with the Phusion enzyme from the genomic DNA of rice using promoter-specific primers (Supplemental Table S1). The PCR product was ligated into pJet1.2 intermediate plasmid following the instruction of CloneJET PCR Cloning Kit (K1232; Thermo Scientific). The *pOsZAS2* sequence was amplified from the pJet1.2 plasmid with specific primers (Supplemental Table S1) and cloned into the pENTR/D-TOPO plasmid. Then, OsZAS2:pENTR/D-TOPO were inserted into pMDC162 (Curtis and Grossniklaus, 2003) by Gateway cloning.

Rice transformation was conducted according to Hiei and Komari (2008). The mutations of transformed lines were analyzed by PCR using a Thermo Scientific Phire Plant Direct PCR Master Mix Kit. Gene-specific primers (Supplemental Table S1) were used for PCR amplification to detect the mutation sites. Then, PCR products were cleaned up using ExoSAP-IT PCR Product Cleanup Reagent and submitted to the Sanger sequencing core lab team, at KAUST. The Sanger sequencing data (abi file) were analyzed following the instruction of DSDecode (Degenerate Sequence Decode; http://skl.scau.edu.cn/dsdecode/). Three independent homozygote mutant lines were identified and grown until T3 generation.

### Metabolite quantification

For quantification, plant material was lyophilized with freeze-dryer and ground with Geno Grinder 2010. D_3_-zaxinone (customized synthesis; Buchem B.V., Apeldoorn, the Netherlands) was used as an internal standard. Zaxinone was extracted according to Wang et al. (2019). SLs were extracted from the root tissues as described by Mi et al. (2018). SL extraction from the root exudates was performed according to Wang et al. (2022). In the final step, the dried extract was dissolved in 110 μL of acetonitrile: water (90:10, v:v) and filtered through a 0.22-μm filter for LC–MS/MS analysis. The samples were run on UHPLC- Triple-Stage Quadrupole Mass Spectrometer (TSQ-Altis) with parameters as described in Wang et al. (2022).

### *Striga* seed germination bioassays

*Striga* seeds were preconditioned as described by Jamil et al. (2019). After 10 days, the preconditioned *Striga* seeds were treated with rice root extracts and exudates, using 50 μL per disc (*n* = 3–6). Root extracts were prepared following the above described SL extraction method. For treatment, 5 μL of root extracts were diluted in 400 μL of MilliQ water before application. Root exudates were collected following the above described protocol. For treatment, 200 μL of SL enriched solution was diluted in 1,800 μL of MilliQ water before application. The discs were also treated with water and GR24 (1 μM) as negative and positive control, respectively. The plates were sealed with parafilm and incubated at 30°C for 24 h. The discs were scanned in a microscope and germinated and nongerminated seeds were counted from these scanned images by using the software SeedQuant (Braguy et al., 2021) to calculate the percentage of germination.

### RT-qPCR analysis

Rice tissues were ground and homogenized in liquid nitrogen, and total RNA was isolated using a Direct-zol RNA Miniprep Plus Kit following the manufacturer’s instructions (ZYMO RESEARCH, Irvine, CA, USA). Briefly, a 1-μg RNA sample was reverse transcribed using an iScript cDNA Synthesis Kit (BIO-RAD Laboratories Inc., 2000 Alfred Nobel Drive, Hercules, CA, USA). The RT-qPCR was performed using SYBR Green Master Mix (Applied Biosystems, Waltham, MA, USA; http://www.lifetechnologies.com) in a CFX384 Touch Real-Time PCR Detection System (BIO-RAD Laboratories, Inc., 2000 Alfred Nobel Drive, Hercules, CA, USA). Primer-BLAST webserver (Ye et al., 2012) was used to design the gene-specific RT-qPCR primers (Supplemental Table S1) and Ubiquitin was used as an internal control. The relative gene expression level was calculated according to 2^Δ^CT method.

### In vitro assays

The OsZAS2 cDNA was amplified using primers listed in Supplemental Table S1 and cloned into the pET-His6 MBP N10 TEV LIC cloning vector (2C-T vector; http://www.addgene.org/29706/) with MBP tags at the N-terminus. OsZAS2-MBP construct transformed into the BL21 Rosetta *E. coli* cells. A single colony of the transformed *E. coli* was cultured overnight and 0.5 mL of this culture was inoculated into 50-mL liquid media and grown at 37°C to OD (optical density) 0.6 at 600 nm. Then, bacteria were induced by IPTG (150 μM final concentration) and kept shaking at 28°C for 4 h. Cells were harvested by centrifugation and resuspended in lysis buffer (sodium phosphate buffer pH 8 containing 1% (v/v) Triton X-100 and 10 mM of dithiothreitol, lysozyme (1 mg mL^−1^)) and incubated on ice for 30 min. Next, the crude lysate was sonicated and centrifuged at 12,000 rpm and 4°C for 10 min, and the supernatant containing the protein was collected for in vitro incubation with the substrate. Synthetic substrates were purchased from Buchem B. V. (Apeldoorn, the Netherlands). Substrates were prepared according to Wang et al. (2019). Dried substrates were resuspended in 0.4% (v/v) Triton X-100 dissolved in ethanol. The mixture was then dried using a vacuum centrifuge to produce an apocarotenoid-containing gel. The gel was resuspended in incubation buffer (2-mM tris 2-carboxyethylphosphine, 0.4-mM FeSO_4_, and 2-mg/mL catalase in 200-mM Hepes/NaOH, pH 8). OsZAS2 crude cell lysate, that is, 50 μL of the soluble fraction of overexpressing cells, was added to the assay. The assay was incubated for 4 h under shaking at 140 rpm at 28°C in dark. The reaction was stopped by adding two volumes of acetone and the lipophilic compounds were separated by partition extraction with petroleum ether: diethyl ether 1: 4 (v/v), dried, and resuspended in methanol for HPLC (High Performance Liquid Chromatography) analysis. For this purpose, we used an Ultimate 3000 UHPLC system and a YMC Carotenoid C30 column (150 × 3.0 mm, 5 μm) following the parameters described in Wang et al. (2019). This experiment was repeated at least 3 times.

### Subcellular localization

The *35S::OsZAS2:mNeongreen* was constructed by amplifying the coding sequence of *OsZAS2* using specific primers (Supplemental Table S1). The PCR product was sub-cloned into the pDONR221 entry vector by BP recombination reaction. Then, the *OsZAS2::pDONR221* fragment was fused into pB7FWG2,0 by Gateway cloning. The construct was introduced into *A. tumefaciens* strain GV3101 by electroporation. *Nicotiana benthamiana* infiltration was performed as described by Aljedaani et al. (2021). *35S::OsZAS2-mNeongreen*, membrane protein marker (*35S::Lit6BmTurquoise2)* and p19 helper plasmid were co-infiltrated into the abaxial leave side of *N. benthamiana*. The fluorescence expression was checked 3-day postinfiltration by the confocal microscope. Leaf tissues of the infiltrated *N. benthamiana* was mounted with water on microscope slides and visualized by using a high-resolution laser confocal microscope (STELLARIS 8 FALCON, Leica). Images were acquired using an HC PL APO CS2 63x/1.20 WATER immersion objective, with 512 × 512 pixel resolution with a line average of 8. The mNeongreeen was excited with a 488 laser, and emission was collected at 500–558 nm. The laser was with 40% intensity, the gain was 180 gain and the pinhole was 1 air unit (AU) pinhole. The mTurquoise2 was excited with a 440 laser and the emission was collected at 445–479. The laser power was 12% intensity. The gain was 74 and the pinhole was 1 AU. This experiment repeated at least 3 times.

### Mycorrhization

Rice seeds of WT cv. DJ, *Oszas2* (*Oszas2-d* and *Oszas2-a*) were germinated in pots containing a sand and incubated for 10 days in a growth chamber under a 14-h light (23°C)/10-h dark (21°C). All genotypes were colonized with approximately 1,000 sterile spores of *Rhizophagus irregularis* DAOM 197198 (Agronutrition, Labège, France). Mycorrhizal plants were grown in sand and watered with a modified Long-Ashton solution containing 3.2-μM Na_2_HPO_4_·12H_2_O and kept in a growth chamber as described before. WT and *Oszas2* mutant plants were sampled at 14 and 50 dpi. To analyze *OsZAS2* gene expression profiles WT plants were inoculated with a fungal inoculum of *Funneliformis mosseae* (BEG 12, MycAgroLab, France) mixed (25% [w/v]) with sterile quartz. Nonmycorrhizal and mycorrhizal plants were sampled at 7 and 21 dpi. For all experiments, mycorrhizal roots were stained with cotton blue, and the level of mycorrhizal colonization was assessed according to Trouvelot et al. (1986) using MYCOCALC (http://www2.dijon.inra.fr/mychintec/Mycocalc-prg/download.html). For molecular analyses, roots were immediately frozen in liquid nitrogen and stored at −80°C. This experiment repeated at least 2 times.

### In situ hybridization and GUS staining

For sample preparation and embedding, rice roots were fixed in 4% (v/v) paraformaldehyde in phosphate-buffered saline (PBS; 130-mM NaCl; 7-mM Na_2_HPO_4_; 3-mM NaH_2_PO_4_, pH 7.4) overnight at 4°C. To facilitate the fixation, samples were placed under vacuum for the first 15–30 min. Then the tissues were dehydrated in successive steps, each of 30–60 min duration, in 30%, 50%, 70%, 80%, 95%, and 100% (v/v) ethanol and 100% (v/v) Neo-Clear (Xylene substitute; Sigma-Aldrich, St Louis, MO, USA). Finally, samples were embedded in paraffin wax (Paraplast plus; Sigma) at 60°C. Sections of 7–8 μm were then transferred to slides treated with 100 mg mL^−1^ poly-L-Lys (Sigma) and dried on a warm plate at 40°C overnight. In parallel, DIG-labeled RNA probes were synthesized starting with 1 μg of PCR-obtained template (Langdale, 1993). DIG-labeled riboprobes (antisense and sense probes) were produced with DIG-UTP by in vitro transcription using the *Sp6* and *T7* promoters, according to the manufacturer’s protocol (RNA-labeling kit; Roche, Basel, Switzerland). The sections were treated as follows: they were de-paraffinized in Neo-Clear, rehydrated through an ethanol series, treated with 0.2-M HCl for 20 min, washed in sterile water for 5 min, incubated in 2× SSC for 10 min, washed in sterile water for 5 min, incubated with proteinase K (1 mg mL^−1^ in 100-mM Tris–HCl, pH 8.0, 50-mM EDTA; Roche) at 37°C for 30 min, washed briefly in PBS, and then treated with 0.2% (w/v) Glycine in PBS for 5 min. After two rinses in PBS, slides were incubated in 4% paraformaldehyde in PBS for 20 min, washed in PBS (2, 3, and 5 min), and then dehydrated in an ethanol series from 30% to 100% (v/v). Hybridizations were carried out overnight at 55°C with denatured DIG-labeled RNA probes in 50% (v/v) formamide, 20×SSC, 20% (w/v) SDS (Sodium dodecyl sulfate), 50-mg mL^−1^ tRNA, 40- μg mL^−1^ Salmon Sperm DNA. Slides were then washed twice in 1× SSC, 0.1% (w/v) SDS at room temperature, and rinsed with 0.2× SSC, 0.1% (w/v) SDS at 55°C (2, 3, and 10 min). After rinsing with 2× SSC for 5 min at room temperature, the nonspecifically bound DIG-labeled probe was removed by incubating in 10-mg mL^−1^ RNase A in 2× SSC at 37°C for 30 min. Slides were then rinsed twice in 2% (v/v) SSC before proceeding to the next stage. The hybridized probe was detected using an alkaline phosphatase antibody conjugate (Roche). After rinsing in TBS (100-mM Tris–HCl, pH 7.5, 400-mM NaCl) for 5 min, slides were treated with 0.5% (w/v) blocking reagent in TBS (Tris Buffered Saline) for 1 h, incubated for 2 h with the anti-DIG alkaline phosphatase conjugate diluted 1:500 in 0.5% (v/v) Bovine Serum Albumin Fraction V in TBS, and then washed in TBS (3 × 5 min). Color development was carried out according to Torres et al. (1995). The color reaction was stopped by washing in distilled water, and the sections were then dehydrated through an ethanol series, deparaffinized in Neo-Clear, and mounted in Neomount (Merck, Kenilworth, NJ, USA) (Balestrini et al., 1997).

The GUS assay was performed on roots of *pOsZAS2:GUS-L11* and *L18* colonized by *F. mosseae* and sampled at 35 dpi. Rice mycorrhizal root segments were cut and placed in single wells of a Multiwell plate and covered with freshly prepared GUS buffer (0.1-M sodium phosphate buffer pH 7.0, 5-mM K_4_Fe(CN)_6_, 5-mM K_3_Fe(CN)_6,_ 0.3% (v/v) Triton X, 0.3% (w/v) x-Glc). To improve buffer penetration into the root segments, they were placed under vacuum for 10 min. Finally, samples were incubated at 37°C for 16 h in the dark, de-stained with 70% (v/v) ethanol, and observed under an optical microscope (Nikon Eclipse E300).

### Accession numbers

The cDNA and promoter sequence of rice ZAS2 is available in NCBI under the accession number LOC107275952. Accessions of SL biosynthetic genes; LOC_Os11g37650 (OsD27), LOC_Os04g46470 (OsCCD7), LOC_Os01g54270 (OsCCD8), and LOC_Os01g50520 (OsCO).

## Supplemental data

The following materials are available in the online version of this article.

**Supplemental Figure S1.** Structures of carotenoids and apocarotenoids used as substrates in ZAS2 in vitro and vivo assays.

**Supplemental Figure S2.** *OsZAS2* expression during AM establishment.

**Supplemental Figure S3.** Relative content of 4DO after zaxinone (5 μM) treatment in root tissue and exudate of Wt and *Oszas2* mutant.

**Supplemental Figure S4.** Zaxinone quantification and *OsZAS* genes expression analysis in *Oszas2* mutants under low Pi conditions.

**Supplemental Figure S5.** Truncated amino acid sequences of *Oszas2* mutant lines after CRISPR-Cas9 genome editing.

**Supplemental Figure S6.** gRNA targets of OsZAS2 were fused to tRNA sequences (Xie et al., 2015).

**Supplemental File S1.** Clustal alignment of ZAS members.

**Supplemental Table S1.** Primer sequences used in this study.

**Supplemental Table S2.** Distribution of ZAS members across monocot and dicot plants.

**Supplemental Table S3.** Protein accessions of ZAS members in different organisms.

## Supplementary Material

kiac472_Supplementary_Data

## Notes

S.A.B. and A.A. conceived and designed the research. S.A.B. supervised the experiments. A.A. generated transgenic lines with the help of CR. C.V., V.F., R.B., and L.L. planned and performed the mycorrhization experiments. M.J. and A.A. conducted the Striga seed germination assays. A.B. performed the in vitro assays. A.A. characterized the transgenic lines with help of M.J. and L.B. J.Y.W., K.X.L., and A.A. performed the metabolite analysis. F.A. performed the subcellular localization experiment. I.B. supervised the subcellular localization and microscopy experiments. A.A. analyzed the data and generated the figures and wrote the manuscript with the help of J.Y.W., V.F., C.V., L.L., and F.A. S.A.B. edited and approved the article.

The author responsible for distribution of materials integral to the findings presented in this article in accordance with the policy described in the Instructions for Authors (https://academic.oup.com/plphys/pages/general-instructions) is Salim Al-Babili (salim.babili@kaust.edu.sa).
