# Supplementary material for: ZAXINONE SYNTHASE 2 regulates growth and arbuscular mycorrhizal symbiosis in rice
Source: Plant Physiol. 2022 Oct 12;191(1):382–99. doi: 10.1093/plphys/kiac472 (PMC9806602; doi:10.1093/plphys/kiac472)
Supplement: kiac472_Supplementary_Data [file kiac472_supplementary_data.pdf]

Supplementary Data

Article title: *ZAXINONE SYNTHASE 2* regulates growth and arbuscular mycorrhizal symbiosis in rice

Authors: Abdugaffor Ablazov[1,2†], Cristina Votta[3†], Valentina Fiorilli[3], Jian You Wang[1], Fatimah Aljedaani[2,4], Muhammad Jamil[1], Aparna Balakrishna[1], Raffaella Balestrini[5], Kit Xi Liew[1], Chakravarthy Rajan[1], Lamis Berqdar[1], Ikram Blilou[2,4], Luisa Lanfranco[3], Salim Al-Babili[1,2*]

The following Supporting Data is available for this article:

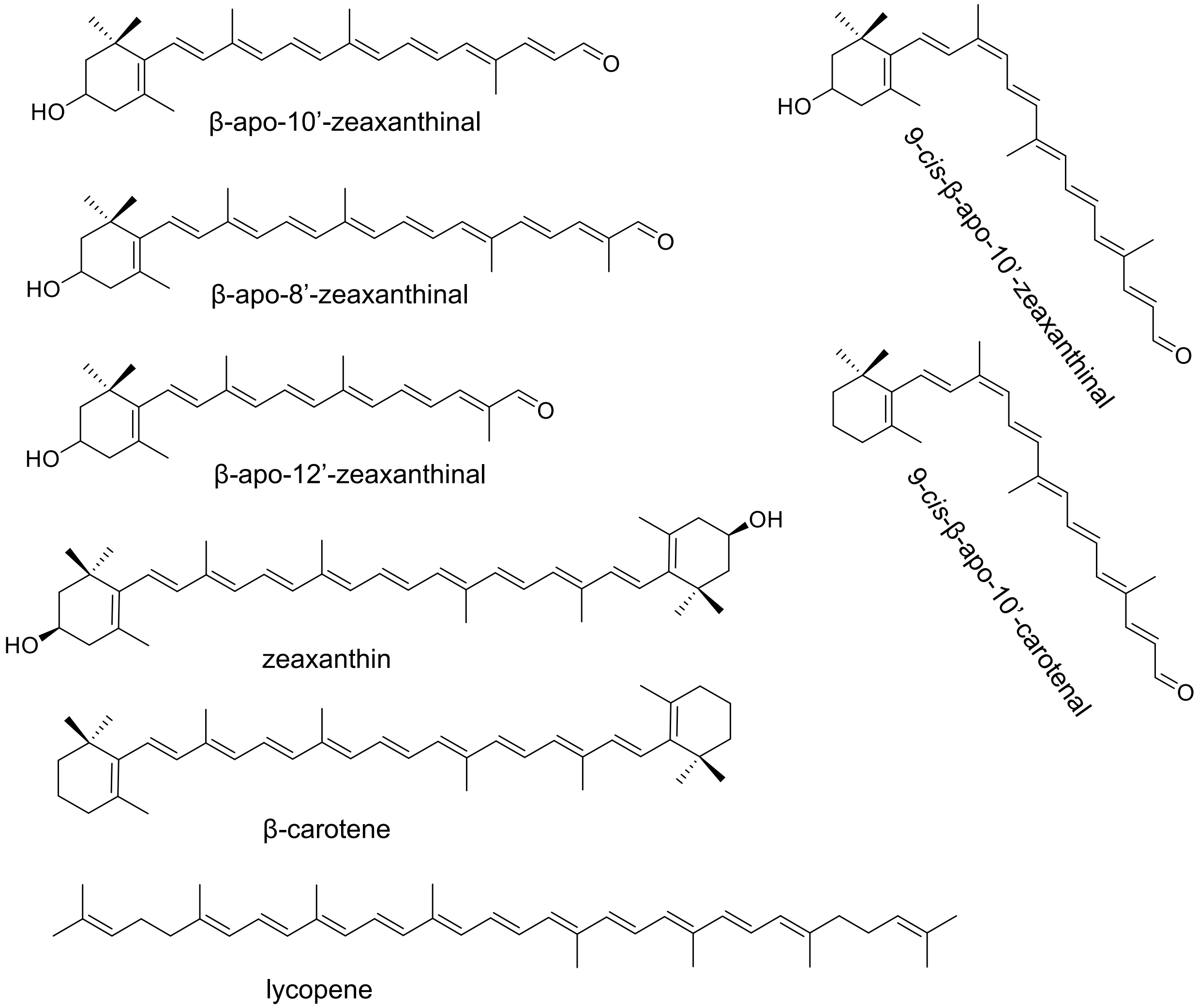

Supplemental Figure S1. Structures of carotenoids and apocarotenoids used as substrates in OsZAS2 *in vitro* and *vivo* assays.

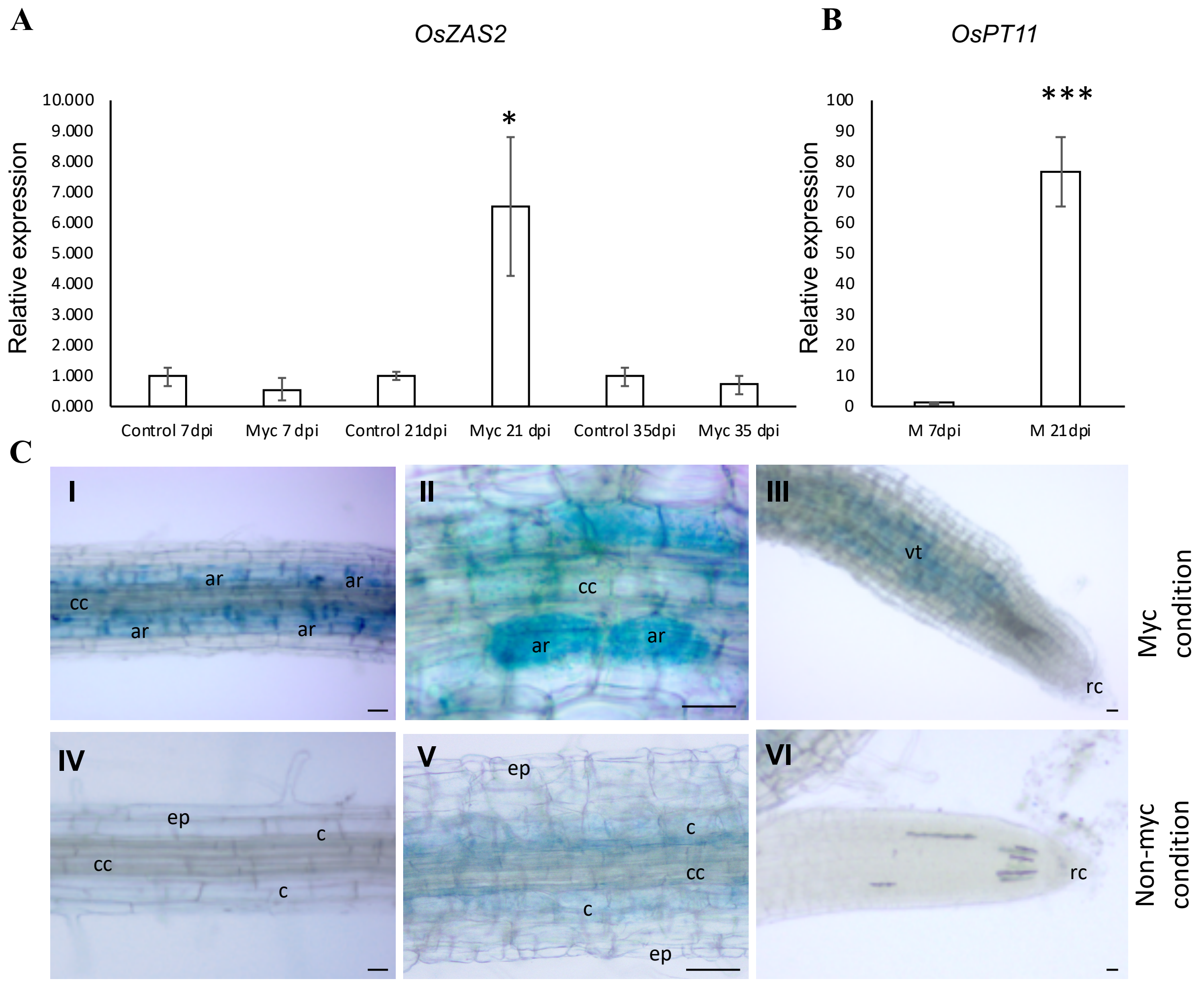

Supplemental Figure S2. *OsZAS2* expression during arbuscular mycorrhizal (AM) establishment. A, *OsZAS2* expression pattern at a different stage of mycorrhizal colonization (*R. irregularis*). B, *OsPT11 (*AM marker gene*)* expression pattern at a different stage of mycorrhizal colonization (*R. irregularis*). C, GUS staining analysis of roots of *pZAS2:GUS-L11* reporter line inoculated (I, II, III) for 35 days with *F. mosseae* and non-inoculated (IV, V, VI). cc, central cylinder; c, non-colonized cortical cells; ep, epidermal cells; vt, vascular tissue; ar, arbuscule containing cells; rc, root cap; dpi, day post-inoculation. Bars in all panels correspond to 50 µm. Values in (A-B) are means ±SD (n ≥ 3) and student's t-test was applied for the statistical analysis (*P ≤ 0.05).

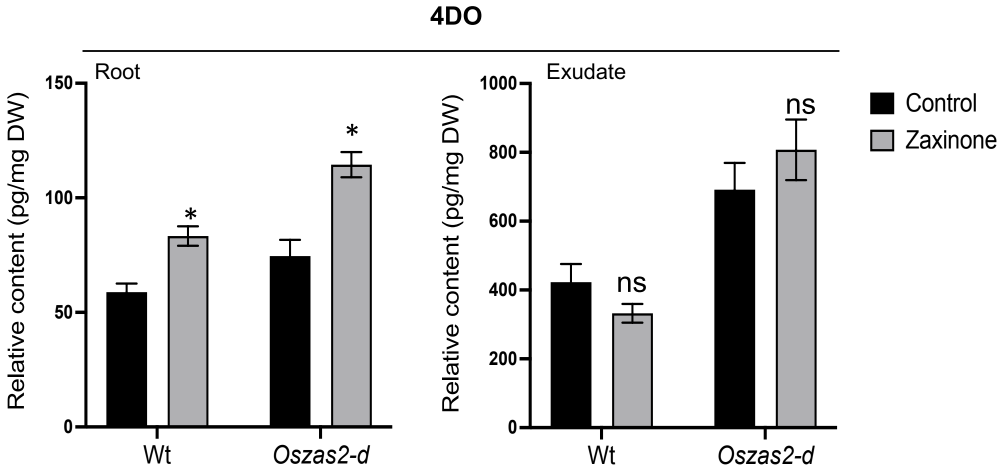

Supplemental Figure S3. Relative content of 4DO after zaxinone (5 μM) treatment in root tissue and exudate of Wt and *Oszas2* mutant. DW, dry weight. Values are means ±SD (n ≥ 5). Student's t-test was applied for the statistical analysis (*P ≤ 0.05; **P ≤ 0.01; ns: not significant).

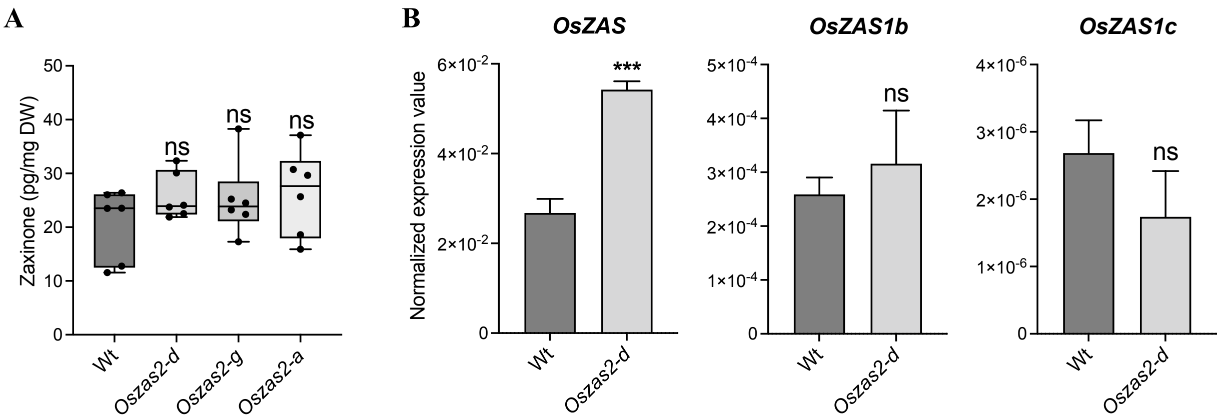

Supplemental Figure S4. Zaxinone quantification and *OsZAS* genes expression analysis in *Oszas2* mutants under low Pi conditions. A, Quantification of zaxinone content in Wt and *Oszas2* mutants roots under low Pi condition. Boxes in boxplots represent the median, first and third quartile. The minimum and maximum values are showed with the length of the whiskers. Dots represent the biological replicates. B, *OsZAS* genes expression analysis in Wt and *Oszas2-d* under low Pi condition. Values in (A-B) are means ±SD ($n \geq 3$) and student's t-test was applied for the statistical analysis (***$P \leq 0.001$; ns, not significant).

**Ref (Wt):**	MFRAGGCSSQVTRCIHLGKSPADQASSLSPPNAVKVQSSVTGWNKQPLMQELTKALKSVSSDLLDRFIDSVYKFSEQPYLNE/---/PQRVPYGFHGNFFYK* 593 aa
Oszas2-d:	MFRAGGCSSQVIHLGKSPADQASSLSPPNAVKVQSSVTGWNKQPLMQELTKALKSVSSAGPIHRLRVQVF* 71 aa
Oszas2-g:	MFRAGGCIHLGKSPADQASSLSPPNAVKVQSSVTGWNKQPLMQELTKALKSVSSDLLGASSTPCTSFLSSLI* 73 aa
Oszas2-a:	MFRAGGCSSQMHSSREEPS* 20 aa

Supplemental Figure S5. Truncated amino acid sequences of *Oszas2* mutant lines after CRISPR-Cas9 genome editing. **aa, amino acid.**

OsZAS2 (tRNA-gRNA1-tRNA-gRNA2)

Standard synthesis of *OsZAS2* (tRNA-gRNA1 -tRNA-gRNA2), including added 5'UTR (GGATGGGCAGTCTGGGCA) and 3'UTR (GTTTGCTGTCGCTCATCC) sequences, with sequence verification and custom cloning into pRGEB32 (Kanamycin) via 5’BsaI and 3’BsaI; delivered as a mini-scale DNA sample.

OsZAS2 (tRNA-gRNA1-tRNA-gRNA2)|Length: 391 |Vector: pRGEB32

GGATGGGCAGTCTGGGCAAACAAAGCACCAGTGGTCTAGTGGTAGAATAGTACCCTGCCACGGTACAGACCCGGGTTC
GATTCCCGGCTGGTGCAGATGAATGCATCTAGTGACCGTTTTAGAGCTAGAAATAGCAAGTTAAAATAAGGCTAGTCCGT
TATCAACTTGAAAAAGTGGCACCGAGTCGGTGCAACAAAGCACCAGTGGTCTAGTGGTAGAATAGTACCCTGCCACGG
TACAGACCCGGGTTCGATTCCCGGCTGGTGCAATCAGTCTCTTCAGATCTGCGTTTTAGAGCTAGAAATAGCAAGTTAAA
ATAAGGCTAGTCCGTTATCAACTTGAAAAAGTGGCACCGAGTCGGTGCTTTTTTTTTGTTTGCTGTCGCTCATCC

The sequences are annotated as follows:
Fok I site: green background and restriction site underlined
gRNA scaffold: yellow background
pre-tRNA: turquoise background
gRNA spacer: red letters

Supplemental Figure S6. gRNA targets of OsZAS2 were fused to tRNA sequences (Xie et al., 2015).

Supplemental File S1. Clustal alignment of ZAS members.

```
                                                  20                  40                  60                  80
NaZAS4      MTFCSYTFQ---VN------------------------------------------------------------------ 11
NtaZAS4a    MAFCSYTFQ---VN------------------------------------------------------------------ 11
NtaZAS4a    MAFCSYTFQ---VN------------------------------------------------------------------ 11
NtoZAS4     -------------------------------------------------------------------------------- –
SlZAS4      MAFCSYTCK---VN------------------------------------------------------------------ 11
SpZAS4      MAFCSYTRK---VN------------------------------------------------------------------ 11
StZAS4b     MALCSYTCK---VN------------------------------------------------------------------ 11
StZAS4a     MALRSYTFK---VN------------------------------------------------------------------ 11
SpZAS3b     MAAYIYACQ---VN------------------------------------------------------------------ 11
SlZAS3b     MATYIYACQ---VN------------------------------------------------------------------ 11
StZAS3b     -------------------------------------------------------------------------------- –
SpZAS3a     MAVCSYTCQ---VN------------------------------------------------------------------ 11
SlZAS3a     M------------------------------------------------------------------------------- 1
StZAS3a     M------------------------------------------------------------------------------- 1
CanZAS3     MVACIYACQ---VN------------------------------------------------------------------ 11
NaZAS5      MAAYSYAL-QVNKC------------------------------------------------------------------ 13
NtaZAS5     M------------------------------------------------------------------------------- 1
StZAS5      MTANSYALLQVVTK------------------------------------------------------------------ 14
BdZAS1a     MCYKLRSNSSHDCRKIRRVSI------------------------RML--TTSSPRTFRPSRTC-RPA---FSLSARTSS 50
AtaZAS1a    MEV---NRPPHDCRRIRLCHYQKS---------------------RMLKATTSPPNALRPSRAYYRPA---FSLSATTSS 53
OsZAS       MM----TASLHPC------------------------------------VCKASP--------AFRPA---SSLGARTQP 29
SbZAS1a     MVLKASAS-----------------------------------------FSPSLPLPLSCRINGGRPS-MSMSAGARTAA 38
ZmZAS1      M--KASAP-----------------------------------------FSPSQPL--SCRTHGGRPSSMSMSAGARTAT 35
OsZAS1b     MCIQGDQKR-----------------------CHPCIPF-------NHAYASPAPGHPSPLK--------LLVYLIRKHP 42
AtaZAS1b    MAVCRDTTSNFVGSSVLVLHGITNATNLEAIACREGLSLAQDLMLQNFVITSDSKQVVNDIKRANRGGYGAVIIEIRQCL 80
OsZAS1c     MVVQKEQHY---------------------------------WNTKT--------INHKP-------------------- 19
BdZAS1c     MVVAAYMPVSSLGQ-------------------RMSFPRIRPWPANTRPAATSSPMASVP-------------------- 41
ZmZAS2      MPLLSLPACCGYDAMFRAGLKPTTSSSRCRSSRLIRSHGGAPDHAPP-FT------------------------------ 49
SbZAS2a     M--------------FRAGLKPTTTSS---SSRCIHSRR-APDHALP-FN------------------------------ 31
SbZAS2b     M--------------FRDGLKPTNSCR----CRCIRSHRAPADHALPCFT------------------------------ 32
OsZAS2      M--------------FRAG------GCSSQVTRCIHLGKSPADQA-SSLS------------------------------ 29
ObZAS2      M--------------FRAA------GCRSQVTRCIHLGKG-TDRS-SSLS------------------------------ 28
AcZAS2      MNSMSMIK-----------------------ARSIH--KSPPT---STLT------------------------------ 22
Consensus   MA--SYT-------------------------------------------------------------------------
Conservation (100% / 0%)

                                                 100                 120                 140                 160
NaZAS4      -----CSYQRPSIPSKVQDVKVSISSALNP-LSRDLVHFPIVAADIPKAI-KETSFKLLDAFVDLVFEFVDQPLLPSQSN 84
NtaZAS4a    -----CSYQRPSIPSKVQDLKVSISSALNP-LSRDLLHFPIVAADIPKAI-KETSFKLLDAFVDLVFEFVDQPLLPSQSN 84
NtaZAS4a    -----CSFQRPSVPSKVQDLRGSISSALNP-ISRELVHFPIV--NIPKAI-KETSFKLLDAFVDLAFEFVDQPLLPSQSN 82
NtoZAS4     --------------------------------------------------------------------FVDQPLLPSQSN 12
SlZAS4      -----CFFQRPSVPSKVEDLKDSISSALKP-FSRDLVHFSIVA-DIPKAV-KETSIKLLDAFVDSVFEFVDQPLLPSQSN 83
SpZAS4      -----CFFQRPSVPSKVEDLKASISSALKP-FSRDLVHFSIVA-DIPKAV-KETSIKLLHAFVDSVFEFVDQPLLPSQSN 83
StZAS4b     -----CSFQRPSIPSKVEDLKDSISSALKP-FSRDLVHFSIVA-DIPKVV-KETSFKLLDAFVDFVFEFVDQPLLPSQSN 83
StZAS4a     -----CSIQRPSIPTKDEDLKASISSALKP-FSRNLVHFPVVA-DIPKVV-KETSIKLLDAFVDLVFEFVDQPLLPSQSN 83
SpZAS3b     -----YCCLKKPLIPKFKDLRASVSSTFKP-FLSNLEN-----LDVQKVI-KEISFKLLDNFVDFTFEFLDQPLLPSQSN 79
SlZAS3b     -----FCCLKKPLIPKFKDLRASVSSTFKP-FLSNLEN-----LDVQKVI-KEISFKLLDNFVDFTFEFVDQPLLPSQSN 79
StZAS3b     -----------------------------------------------------------------------QPLLPSQSN 9
SpZAS3a     -----YCCLQRHVIPKFKDLRASVSSNFKP-FLTNLEHFPLI-MDVPKVILKEVIFKLLDNFIDLTFEFVDQPLSPSQSN 84
SlZAS3a     --------------------------------------------DVPKVILKEVIFKLLDNFIDLTFEFVDQPLSPSQSN 37
StZAS3a     --------------------------------------------DGMKLGIGAISGRNL--------EVGWRPPIRAEHK 29
CanZAS3     -----YCCSKKPLIPKFKDLKASISSTLKP-FLTNLEHFPLI-MDVPKAT-KETSFKLLDTFVDLIFEFVDQPLLPSKSN 83
NaZAS5      -----SSPQRPSLPQKIEHFSNPLSTSFKP-LLRGLDQFPNISVDVTKAI-KKTSCKLLDAFVDFIFEFVGQPLLPSQRN 86
NtaZAS5     -----------------------------------------------------------------VWSIIA--------- 7
StZAS5      -----CSLQRPSLPPKLEHSKNPLSSTFKPILLKELDHFPNISLDVPKAI-KDTSFNLLEAFVDFIYEFVDQPLLPCQSN 88
BdZAS1a     SSITA-----NSKKPLFQELQRQLSLKI----------------DDASQILKNAPHRLLDAIVDSTFKFTAQALLPSESN 109
AtaZAS1a    SSLTS-----NPKKSLIQELRRQLSSTI----------------DDASQVLKNAPQKLLDAIVDSTFKFTHQALLPSESN 112
OsZAS       KSTAT-----NPKRPLFQELQRRLSFRI----------------DEASKALETAKQGLLDALVDSTFKFSDQPMLPSENN 88
SbZAS1a     -SVGT-----SSQKPLLGELLGNLSSKM----------------DRASKALKDVPQRFLDVLVDATFKFTDEALNPSESN 96
ZmZAS1      -SVGT-----SSKRPWFGDLLGRLSSTM----------------DGASRALKDAPQRFLDALVDATFRFTDQALNSAESN 93
OsZAS1b     L---------------EREIQVHLSSKLG----------------EASNAMNSTYQQLLDSFVDHTFTFKCQPLRPTESN 91
AtaZAS1b    LAFNCIITFKGTTANNDADMLAKFAHSLGTC----------DVFNSSSNALNYASQRLLDAFVGTVFTLSHQPLRPTEGN 150
OsZAS1c     ---------------HFKEFQQQLTTKLG----------------EASAAVTMASSLLLDAFVDSTFTFSHQSLRPTESN 68
BdZAS1c     ---------------YFKELQQQITSKLA----------------EASER-------LLDAFVDSTFTFSNQSLRPTESN 83
ZmZAS2      -------------QPNSVKVPPS------PGLKKQ-----ALSVQDMSRALKSLSSDLLQRFVDRAYSFSEEPSL-NEGN 104
SbZAS2a     -------------PPNAIK-----------------------------------------------------------GN 39
SbZAS2b     -------------PPNAIKVPPS------PGLKKQ-----ALILQGISQALKSVSSNLLERFIDRAYRFSEQPSL-NEGN 87
OsZAS2      -------------PPNAVKVQSS-----VTGWNKQ-----PL-MQELTKALKSVSSDLLDRFIDSVYKFSEQPYL-NEGN 84
ObZAS2      -------------PPNAIKVQSS-----VTGWKKQ-----TL-IQELTKTLKSVSLDLLDRFIDSAYKFSEQPFL-NEGN 83
AcZAS2      -------------PHNFISSLSSPFKTLLKEWEKL-----PLRI-EVSNSMKAASLNLVDAFVDWMYKFSDLPAL-IEEN 82
Consensus   -------------PPKFXDLKAS-SS-L-P--------------DXPKAI-KETSFKLLDAFVDSTFEFVDQPLLPSXSN
Conservation (100% / 0%)
```

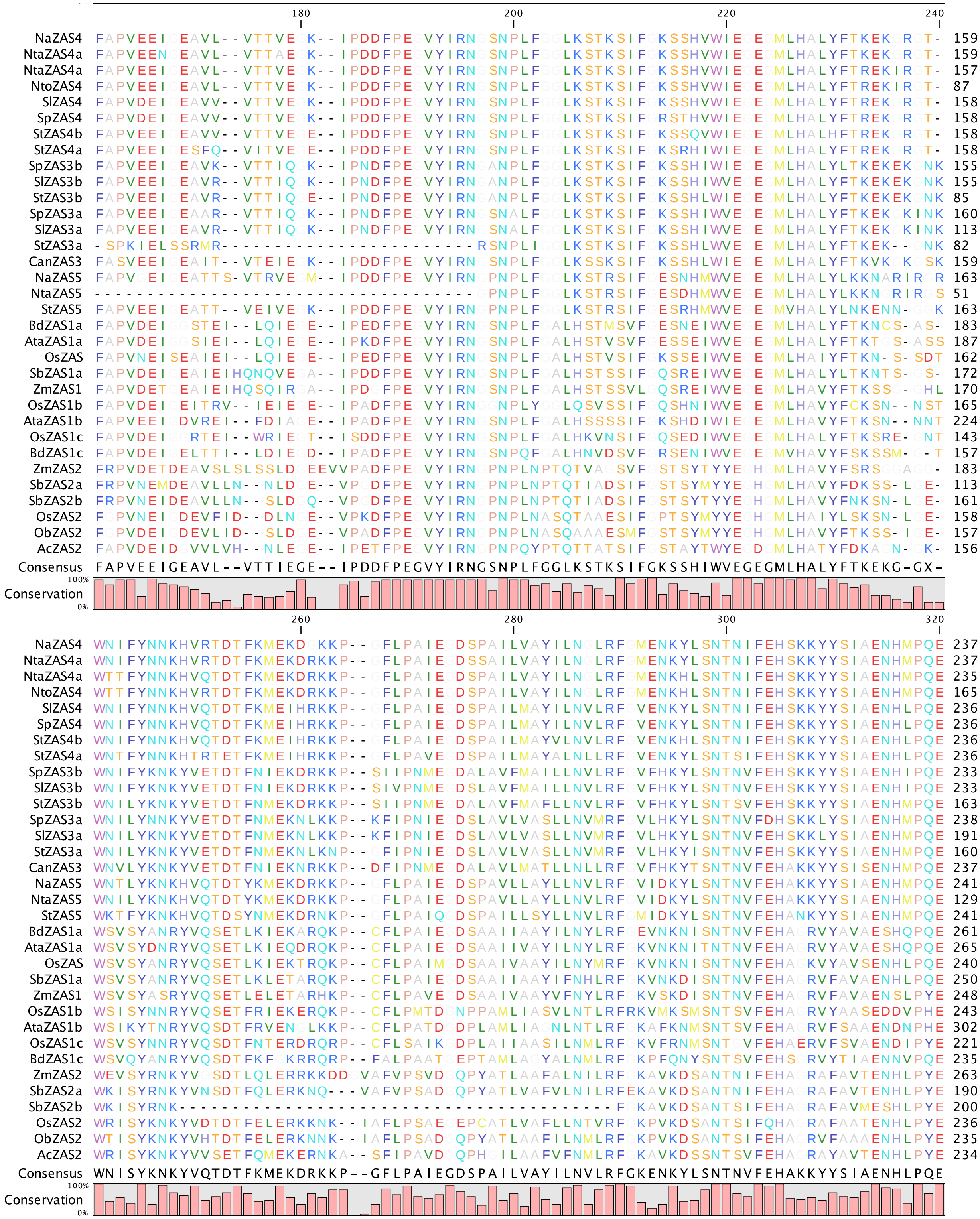
180
200
220
240
NaZAS4
NtaZAS4a
NtaZAS4a
NtoZAS4
SlZAS4
SpZAS4
StZAS4b
StZAS4a
SpZAS3b
SlZAS3b
StZAS3b
SpZAS3a
SlZAS3a
StZAS3a
CanZAS3
NaZAS5
NtaZAS5
StZAS5
BdZAS1a
AtaZAS1a
OsZAS
SbZAS1a
ZmZAS1
OsZAS1b
AtaZAS1b
OsZAS1c
BdZAS1c
ZmZAS2
SbZAS2a
SbZAS2b
OsZAS2
ObZAS2
AcZAS2
Consensus FAPVEEIGEAVL--VTTIEGE--IPDDFPEGVYIRNGSNPLFGGLKSTKSIFGKSSHIWVEGEGMLHALYFTKEKG-GX-
100%
Conservation
0%
260
280
300
320
Consensus WNISYKNKYVQTDTFKMEKDRKKP--GFLPAIEGDSPAILVAYILNVLRFGKENKYLSNTNVFEHAKKYYSIAENHLPQE
100%
Conservation
0%

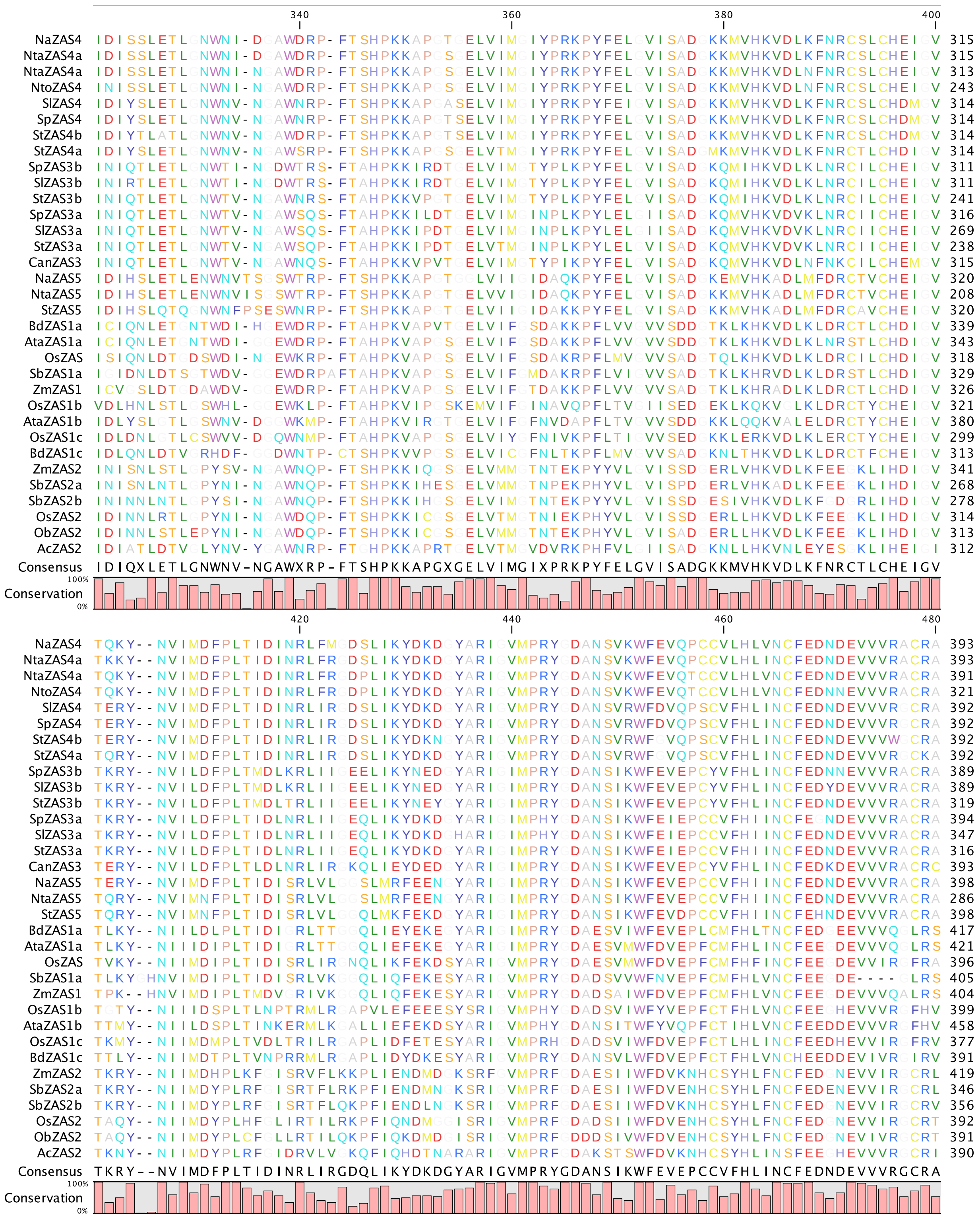

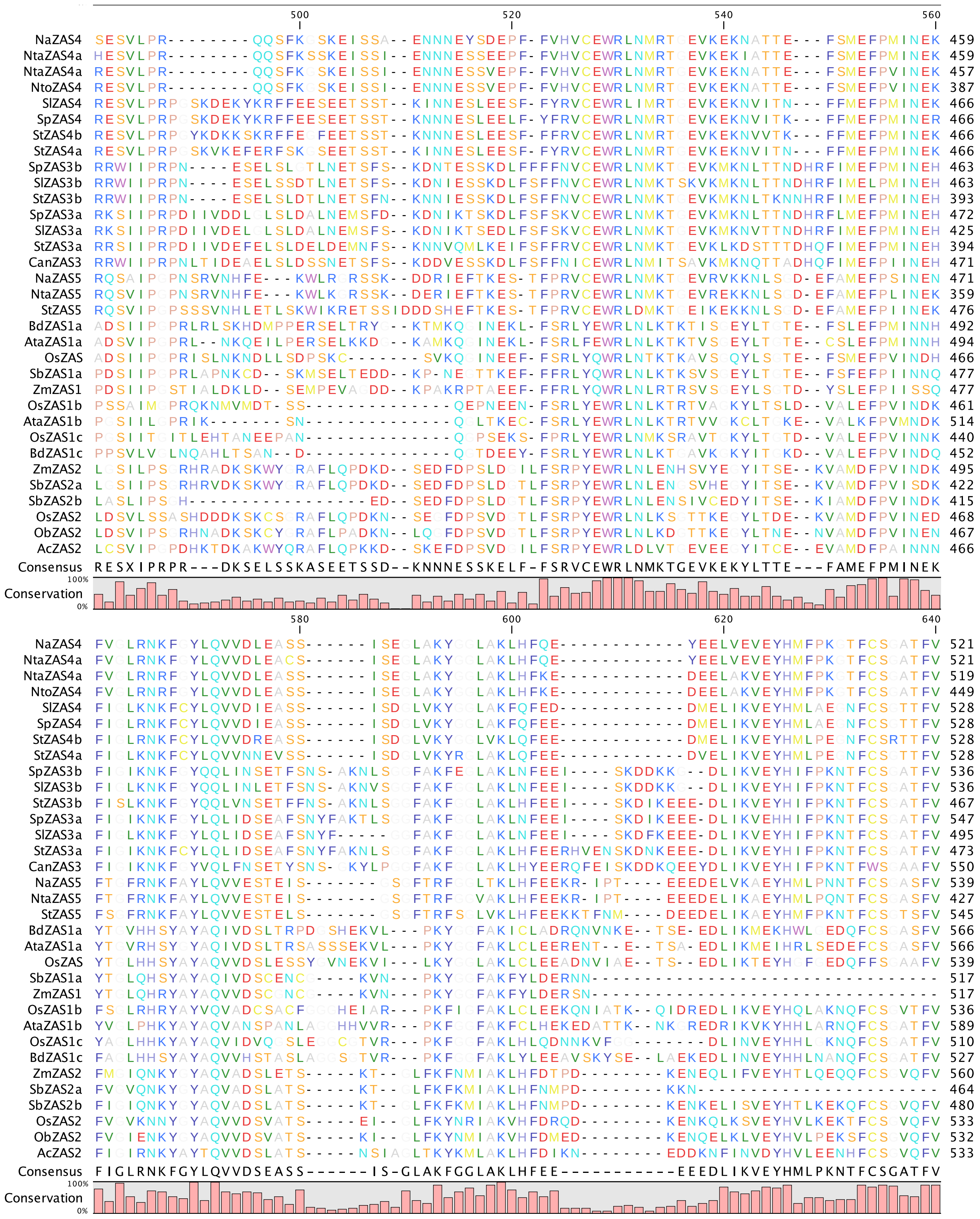
500
520
540
560
580
600
620
640
Consensus
Conservation
100%
0%

```
                       660                 680                 700                 720
NaZAS4     PKTQGTDEDDGWVVTFTHNETTNVSQVYVVDAKSFATQPVAIITLSSRVPYGFH-----GAFMP----------------  580
NtaZAS4a   PKTQGTDENDGWVVTFTHNESTNVSQVYVVDAKNFATQPVAIFTLPSRVPYGFH-----GAFMP----------------  580
NtaZAS4a   PKTQGTDEDDGWVVTFTHNESTNVSQE-----------------------------------------------------  546
NtoZAS4    PKTQGTDEDDGWVVTFTHNESTNVSQVYVVDANNFATQSVATITLPSRVPYGFH-----GAFMP----------------  508
SlZAS4     PKPQGVDEDDGWLVTFLHNENTNVSHVYIVDAKKFATHPITTITLPSRVPYGFH-----GAFMP----------------  587
SpZAS4     PKPQGVDEDDGWLVTFLHNENTNVSHVYIVDAKKFATHPITTITLPNRVPYGFH-----GAFMP----------------  587
StZAS4b    PKPQGVDEDDGWLVTFIHNENTNVSHVYIVDAKNFATHPIATITLPSRVPYGFH-----GAFMP----------------  587
StZAS4a    PKSQGVDEDDGWLVTFTHNENTNVSQVYIVDAKNFATHPIATITLPSRVPYGFH-----GAFMP----------------  587
SpZAS3b    PKLDGVDEDDGWIVAFTHNENTNQSQVYIVDAKNFENEPIAIILLPKRVPYGFH-----GVFMP----------------  595
SlZAS3b    PKLDGVDEDDGWIVAFTHNENTNQSQVYILDAKNFENEPIAIILLPNRVPYGFH-----GVFML----------------  595
StZAS3b    PKLDGVDEDDGWILTFTHNENTNQSQVYIVDAENFESEPIAIILLPNRVPYGFH-----GAFMP----------------  526
SpZAS3a    PKLDGVDEDDGWIVAFTHNENTNQSQVYIIDAKKFGSEAVAIIILPSRVPYGFH-----GAFMS----------------  606
SlZAS3a    PKLDGVDEDDGWIVAFTHNENTNQSQVYIIDAKKFESEAVAIIILPSRVPYGFH-----GAFMS----------------  554
StZAS3a    PKLDGVDEDDGWIVAFTHNENTNQSQVYIIDAKKFESEAVAIIILPSRVPYGFH-----GAFMS----------------  532
CanZAS3    PKVEGVDEDDGWIVAFTHNEDTNQSQVYIIDAKKIESKPVAIITLPSRVPYGFH-----GAFLP----------------  609
NaZAS5     PKPGGVDEDDGWIITFTHNENENISQVYIVDAKKFTNEPVAIITLPSRVPYGFH-----GAFMP----------------  598
NtaZAS5    PKPGGVDEDDGWIITFTHNENENISQVYIVDAKKFTNEPVAIITLPSRVPYGFH-----GAFMP----------------  486
StZAS5     PKPEGIDEDDGWIITFTHNENENISQVCIIDAKKFTKEPVAIITLSSRVPYGFH-----GAFMP----------------  604
BdZAS1a    PRVGGSHEDDGWIISFVHNERTNKSQVHIIDTRRFEGAPVAKITLPQRVPYGFH-----GTFVHTNL-------------  628
AtaZAS1a   PRVGGSHEDDGWIISFVHNERTNTSQVHIIDTQKFEGAPVAKITLPQRVPYGFH-----GTFVHNNIHGHVNTRSEKKLD  641
OsZAS      PRVGGSHEDDGWIISFVHDERSNTSQVHIIDAQTFEGAPVAKIVLPQRVPYGFH-----GTF-RSSLANTMT---------  605
SbZAS1a    ------------------------TEVHIVDAKRFEDAPVAKITLPRRVPYGFH-----GTFISKKL-------------  555
ZmZAS1     ------------------------AEI-----------PGASLVKTQYHWLGKHEFCSGASFVPRA--------------  548
OsZAS1b    PKAAGAHEDDGWIVSFVHDEETNISKVHIIDARNFESEPIAKIILPQRVPYGLH-----GAFIT----KRT---------  598
AtaZAS1b   PKVNGTNEDDGWIVSFVHDEGTNKSKVHIINAQTFEYEPIAKIALPQRVPYGFH-----GAFVS----KST---------  651
OsZAS1c    PKVNGANEDDGWIISFVHDEEINTSQAHVIDAQRFENGPIAKITLPQRVPYGFH-----GTFIPRTTYKKT---------  576
BdZAS1c    PNVNGGHEDHGWIISFVHDEDTNISQAHIINTRRFESEAIAKITLPQRVPYGFH-----GTFIS----KNK---------  589
ZmZAS2     VKENGIDEDDGWLVTYVHDERTNISQVYIIDAKRFSEEPVAKITLPQRVPYGFH-----GNFFYTSNQR-----------  624
SbZAS2a    ----GIDEDDGWIVTYVHDEGTNSSQVYIIDAKRFSEEPVAKITLPQRVPYGFH-----GNFFYTSNQR-----------  524
SbZAS2b    AKKNGIDEDDGWVVTYVHDEGTNVSQVHIIDAKRFSEEPVAKITLPQRVPYGFH-----GNFFYTSNQR-----------  544
OsZAS2     AKENGIDEDDGWIITYVHDELTNVSQVYIIDAKRFAEEPVLKITLPQRVPYGFH-----GNFFY----K-----------  593
ObZAS2     AKKNGIDEDDGWIVTYVHDELTNISQVYIIDAKRFSEEPVVKITLPQRVPYGFH-----GNFVY----K-----------  592
AcZAS2     EKKDAVDEDDGWLISYVHDEKANVSKVYIIDAKRFTEEPVAKITLPQRVPYGFH-----GIFI----SK-----------  593
Consensus  PKVQGVDEDDGWIVTFTHNENTNVSQVYIIDAKXFEXEPVAKITLPQRVPYGFH-----GAFMP----------------
           100%
Conservation
           0%
```

```
NaZAS4     -L----  581
NtaZAS4a   -L----  581
NtaZAS4a   ------  546
NtoZAS4    -S----  509
SlZAS4     -L----  588
SpZAS4     -L----  588
StZAS4b    -L----  588
StZAS4a    -L----  588
SpZAS3b    -FNL--  598
SlZAS3b    -FNL--  598
StZAS3b    -FNL--  529
SpZAS3a    -FN---  608
SlZAS3a    -SN---  556
StZAS3a    -FNIPY  537
CanZAS3    -FNL--  612
NaZAS5     -LE--S  601
NtaZAS5    -LE--S  489
StZAS5     -LT--S  607
BdZAS1a    ------  628
AtaZAS1a   NLKFIY  647
OsZAS      ------  605
SbZAS1a    ----IM  557
ZmZAS1     -----G  549
OsZAS1b    ------  598
AtaZAS1b   ------  651
OsZAS1c    ------  576
BdZAS1c    ------  589
ZmZAS2     ------  624
SbZAS2a    ------  524
SbZAS2b    ------  544
OsZAS2     ------  593
ObZAS2     ------  592
AcZAS2     ------  593
Consensus  ------
           100%
Conservation
           0%
```

Supplemental Table S1 Primer sequences used in this study.

Experiment	Primer name	Sequence (5′–3′)
RT-qPCR analyses	UBQ-Q-F	GCCCAAGAAGAAGATCAAGAAC
	UBQ-Q-R	AGATAACAACGGAAGCATAAAAGTC
	OsD27-Q-F	CTTCCAAGCTACATCCTCAC
	OsD27-Q-R	CCCAACCAACCAAGGAAA
	OsCCD7-Q-F	CAGTCTCCAAGCACAGATG
	OsCCD7-Q-R	GTTCTTTGGCACCTCTAGTT
	OsCCD8-Q-F	TGGCGATATCGATGGTGA
	OsCCD8-Q-R	GACCTCCTCGAACGTCTT
	OsCO-Q-F	ATTGTCAGCGATCCACTTC
	OsCO-Q-R	GCGCCGTTCTTGAAATTG
	OsZAS-Q-F	CAGCCAAAATCCACAGCCAC
	OsZAS-Q-R	AAGCCCCTGTTTTGCTGTCT
	OsZAS1a-Q-F	GACCAAAAACGGTGCCATCC
	OsZAS1a-Q-R	CTCCCTTTCCAGTGGATGCT
	OsZAS1b-Q-F	GCAGCAGCTTACCACCAAAC
	OsZAS1b-Q-R	GTGCGAAATTGCTCTCGGTG
	OsZAS2-Q-F	CATCTAGGGAAGAGCCCAGC
	OsZAS2-Q-R	TGGCTGCTTATTCCACCCAG
	RiEFα-F	GCTATTTTGATCATTGCCGCC
	RiEFα-R	TCATTAAAACGTTCTTCCGACC
	OSPt11-Q-F	GAGAAGTTCCCTGCTTCAAGCA
	OSPt11-Q-R	CATATCCCAGATGAGCGTATCATG
	RiPEIP1-Q-F	AAGAAAGTAAACGTGTGGCT
	RiPEIP1-Q-R	TAACACTCATCTCGGGACTG
	OsRubQ1-F	GGGTTCACAAGTCTGCCTATTTG
	OsRubQ1-R	ACGGGACACGACCAAGGA
Oszas2 mutant screening	gOsZAS2-F	ACAAGCTCGAACTAGCAGGC
	gOsZAS2-R	GTTGCTCGTCGACTTCACAAAT
pOsZAS2:GUS reporter	pOsZAS2-F	TGTACGCGGCTGGCCTATTC
	pOsZAS2-F-CACC	CACCTGTACGCGGCTGGCCTATTC
	pOsZAS2-R	GGCTTGCTCAGGTTATTCGATCC
In situ hybridization	OsZAS2-mid-F	CTCCGCACCCTAGGACCTTA
	OsZAS2-mid-R	TAGGCATCACTCCAATGCGT
	OsZAS2-SP6-R	CTATTTAGGTGACACTATAGACTCCGCACCCTAGGACCTTA
	M13-F	GTAAAACGACGGCCAG
In vitro assay	OsZAS2-CDS-F	ATGTTCAGAGCTGGAGGGT
	OsZAS2-CDS-R	TCACTTGTAGAAAAAATTTCCATG
	OsZAS2-MBP-F	CCAGGGAGCAGCCTCGatgttcagagctggagggtgca
	OsZAS2-MBP-R	GCAAAGCACCGGCCTCGtcacttgtagaaaaaatttccatgaaacccatat
Subcellular localization	OsZAS2-CDS-Box2-F	GGGGACAAGTTTGTACAAAAAAGCAGGCTTAATGTTCAGAGCTGGAGGGTG
	OsZAS2-CDS-Box2-R	GGGGACCACTTTGTACAAGAAAGCTGGGTTCTTGTAGAAAAAATTTCCATGAAACCCATATGGAAC

Supplemental Table S2 Distribution of ZAS members across monocot and dicot plants. Abreviation: SN, shorten name of the species used in phlygenetic trees of this study. Columns from 4th to 8th represent numbers of Clade I-V orthologues in each species' genome. This table adopted from Wang et al., 2019.

SN	Species	RefSeq Assembly Accession	Clade I	Clade II	Clade III	Clade IV	Clade V
Monocot							
Os	*Oryza sativa*	GCF_001433935.1	3	1	0	0	0
Zm	*Zea mays*	GCF_000005005.1	1	1	0	0	0
Ob	*Oryza brachyantha*	GCF_000231095.1	0	1	0	0	0
Bd	*Brachypodium distachyon*	GCF_000005505.2	2	0	0	0	0
Ata	*Aegilops tauschii subsp. tauschii*	GCF_001957025.1	2	0	0	0	0
Sb	*Sorghum bicolor*	GCF_000003195.2	1	2	0	0	0
Ac	*Ananas comosus*	GCF_001540865.1	0	1	0	0	0
Dicot							
Na	*Nicotiana attenuata*	GCF_001879085.1	0	0	0	1	1
Nto	*Nicotiana tomentosiformis*	GCF_000390325.2	0	0	0	1	0
Nta	*Nicotiana tabacum*	GCF_000715135.1	0	0	0	2	1
Can	*Capsicum annuum*	GCF_000710875.1	0	0	1	0	0
St	*Solanum tuberosum*	GCF_000226075.1	0	0	2	2	2
Sp	*Solanum pennellii*	GCF_001406875.1	0	0	2	1	0
Sl	*Solanum lycopersicum*	GCF_000188115.3	0	0	2	1	0
			9	6	7	8	4

Supplemental Table S3 Protein accession of ZAS members in different organisms.

Clades	Gene Symbol	Protein Accession (NCBI)
Clade I	BdZAS1a	XP_003576413.2
	AtaZAS1a	XP_020201457.1
	OsZAS	XP_015611401.1
	SbZAS1a	XP_002462200.1
	ZmZAS1	NP_001152477.1
	OsZAS1b	XP_015648368.1
	AtaZAS1b	XP_020149972.1
	OsZAS1c	XP_015649272.1
	BdZAS1c	XP_010235077.1
Clade II	ZmZAS2	XP_008659260.1
	SbZAS2a	XP_002437880.1
	SbZAS2b	XP_002437880.1
	OsZAS2	XP015641132.1
	ObZAS2	XP_015693936.1
	AcZAS2	XP_020098001.1
Clade III	SpZAS3a	XP_015058049.1
	SlZAS3a	XP_019066509.1
	StZAS3a	XP_015158838.1
	SpZAS3b	XP_015057245.1
	SlZAS3b	XP_004251419.1
	StZAS3b	XP_015158843.1
	CanZAS3	XP_016546962.1
Clade IV	NtoZAS4	XP_009601557.1
	NtaZAS4a	XP_016482181.1
	NaZAS4	XP_019242827.1
	NtaZAS4a	XP_016506620.1
	StZAS4a	XP_006359778.1
	StZAS4b	XP_006359779.1
	SlZAS4	XP_004245397.1
	SpZAS4	XP_015084337.1
Clade V	NaZAS5	XP_019231151.1
	NtaZAS5	XP_016506622.1
	StZAS5	XP_006359776.1
